# Predictive Mortality and Gastric Cancer Risk Using Clinical and Socio-Economic Data: A Nationwide Multicenter Cohort Study

**DOI:** 10.3390/cancers17010030

**Published:** 2024-12-25

**Authors:** Seong Uk Kang, Seung-Joo Nam, Oh Beom Kwon, Inhyeok Yim, Tae-Hoon Kim, Na Young Yeo, Myoung Nam Lim, Woo Jin Kim, Sang Won Park

**Affiliations:** 1 Department of Bigdata, Kangwon National University Hospital, Chuncheon 24289, Republic of Korea; uk99@kangwon.ac.kr; 2 Department of Convergence Security, Kangwon National University, Chuncheon 24341, Republic of Korea; 3 Department of Gastroenterology, Kangwon National University Hospital, Chuncheon 24289, Republic of Korea; pinetrees@daum.net; 4 Department of Pulmonology, Kangwon National University Hospital, Chuncheon 24289, Republic of Korea; shob86@naver.com; 5 Department of Family Medicine, Kangwon National University Hospital, School of Medicine, Kangwon National University, Chuncheon 24289, Republic of Korea; 6 University-Industry Cooperation Foundation, Kangwon National University, Chuncheon 24341, Republic of Korea; blessing0104@naver.com; 7 Department of Medical Big-Data Convergence, Kangwon National University, Chuncheon 24341, Republic of Korea; nayung91@gmail.com; 8 Biomedical Research Institute, Kangwon National University Hospital, Chuncheon 24289, Republic of Korea; lmn99054@kangwon.ac.kr; 9 Department of Internal Medicine, Kangwon National University Hospital, Chuncheon 24289, Republic of Korea; 10 Department of Internal Medicine, School of Medicine, Kangwon National University, Chuncheon 24341, Republic of Korea; 11 Department of Next Generation Information Center, Kangwon National University Hospital, Chuncheon 24289, Republic of Korea; 12 Department of Data Science, Weknew Co., Ltd., Chuncheon 24341, Republic of Korea

**Keywords:** gastric cancer, mortality, cohort study, lifestyle factors, machine learning

## Abstract

**Background/Objectives:** Gastric cancer is a leading cause of cancer-related mortality, particularly in East Asia, with a notable burden in Republic of Korea. This study aimed to construct and develop machine learning models for the prediction of gastric cancer mortality and the identification of risk factors. **Methods:** All data were acquired from the Korean Clinical Data Utilization for Research Excellence by multiple medical centers in South Korea. A total of 23,717 gastric cancer patients were divided into two groups by cause of mortality (all-cause of 2664 and disease-specific of 1620) and investigated. We used comprehensive data integrating clinical, pathological, lifestyle, and socio-economic factors. Cox proportional hazards analysis was conducted to estimate hazard ratios for mortality. Five machine learning models (random forest, gradient boosting machine, XGBoost, light GBM, and cat boosting) were developed to predict mortality. The models were interpreted by SHAP, one of the explainable AI techniques. **Results:** For all-cause mortality, the gradient-boosting machine learning model demonstrated the highest performance with an AUC-ROC of 0.795. For disease-specific mortality, the light GBM model outperformed others, achieving an AUC-ROC of 0.867. Significant predictors included the AJCC7 stage, tumor size, lymph node count, and lifestyle factors such as smoking, drinking, and diabetes. **Conclusions:** This study underscores the importance of integrating both clinical and lifestyle data to enhance mortality prediction accuracy in gastric cancer patients. The findings highlight the need for personalized treatment approaches in the Korean population and emphasize the role of demographic-specific data in predictive modeling.

## Simple Summary

Gastric cancer (GC) affects more than one million and is the fifth most frequently diagnosed cancer and the fourth leading cause of cancer death worldwide. Past studies have usually focused on a limited number of clinical or demographic factors to predict GC prognosis. In contrast, we used twenty-four features, including demographic, laboratory, clinical, and socio-economic information, to predict GC mortality. We investigated two case groups divided by cause of mortality (all-cause and disease-specific) with the construction of six machine learning (ML) models. In addition, the Shapley Additive Explanation (SHAP) method, an explainable artificial intelligence technique, was used. This approach allows us to identify and interpret the key features that have a significant impact on GC mortality. Key predictors of the mortality classification model included occurrence in other organs, age at diagnosis, AJCC7 stage, tumor size, CEA, smoking, and CA19-9. Accurate prediction of mortality and detection of risk factors for GC based on ML might provide opportunities for appropriate therapeutic interventions and decision-making.

## 1. Introduction

Gastric cancer (GC) is a significant global health concern, with more than one million new cases annually. It ranks as the fifth most common cancer diagnosis and the fourth leading cause of cancer-related deaths worldwide [1,2,3]. The incidence of GC is notably higher in regions such as Eastern and Central Asia, as well as Latin and South America, with Korea experiencing a particularly heavy burden [3,4,5,6]. Understanding mortality predictors and identifying key risk factors for GC patients are vital for improving clinical decision-making, optimizing patient care environments, and implementing effective interventions [7].

Traditionally, statistical methods have been used to analyze GC mortality rates. However, the increasing availability of large datasets and the demand for improved risk prediction tools have driven the rapid adoption of machine learning (ML) in this field [8,9,10]. Recent studies utilizing large cohort datasets, such as SEER [11,12], NCDB [13,14,15,16,17], TCGA [18,19,20,21,22,23,24,25,26,27], and GLOBOCAN [28,29,30,31,32,33,34], have advanced understanding of GC prognosis and treatment by integrating demographic, environmental, and lifestyle data. While these studies highlight key risk factors, the applicability of findings from Western datasets to Korean populations is limited due to genetic and environmental differences. Research in Korea has utilized national cancer registry data to perform survival analyses [35] and calculate survival rates based on treatments, including gastrectomy [36]. Accurate GC mortality prediction requires models that integrate clinical, pathological, and social risk factors, such as diet [37,38,39], smoking [40,41], alcohol consumption [40,42,43], and obesity [44,45,46]. Thus, developing comprehensive models that account for both medical and lifestyle variables is critical for precise outcome predictions.

**Figure 1:**
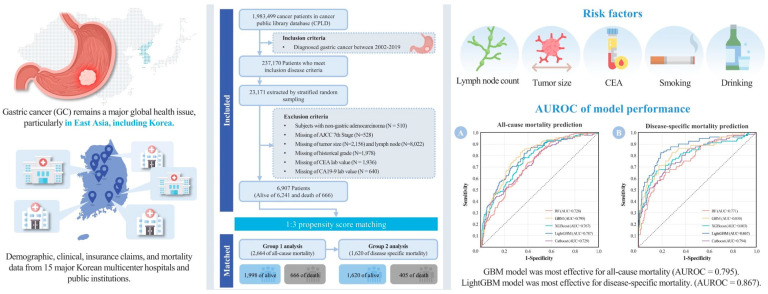
The scheme of study flow.

In this study, we developed robust predictive classification models for GC mortality using extensive data collected from multiple medical centers across South Korea. Key risk factors were identified, and the most optimized ML model suitable for practical clinical application was proposed (Figure 1). Through the application of the SHapley Additive exPlanation (SHAP) method, one of the explainable artificial intelligence (XAI) techniques, we presented a more detailed model result interpretation that overcame the limitations of ML characteristics, which are black-box models.

## 2. Patients and Methods

### 2.1. Patients

#### 2.1.1. Data Source

We utilized data from the Korean Clinical Data Utilization Network for Research Excellence (K-CURE) in South Korea [47]. This cohort was initiated by Ministry of Health and Welfare in South Korea based on Personal Information Protection and Cancer Control Acts. As a representative of South Korea’s cancer data from 15 major hospitals [48], the K-CURE cohort was established, combining data from four major population-based public sources: the Korea National Cancer Incidence Database (KNCI DB) in the Korea Central Cancer Registry (KCCR), cause-of-death data in Statistics Korea, National Health Information Database (NHID) in the National Health Insurance Service (NHIS), and National Health Insurance Research Database (NHIRD) in the Health Insurance Review and Assessment Service (HIRA). The K-CURE contains demographic data such as age, sex, residence, diagnosis date, and cancer type based on the International Classification of Diseases (ICD) 10th edition, SEER summary stage, morphology, and treatment methods for patients with cancer [47,49].

#### 2.1.2. Study Population and Data Collection

**Figure 2:**
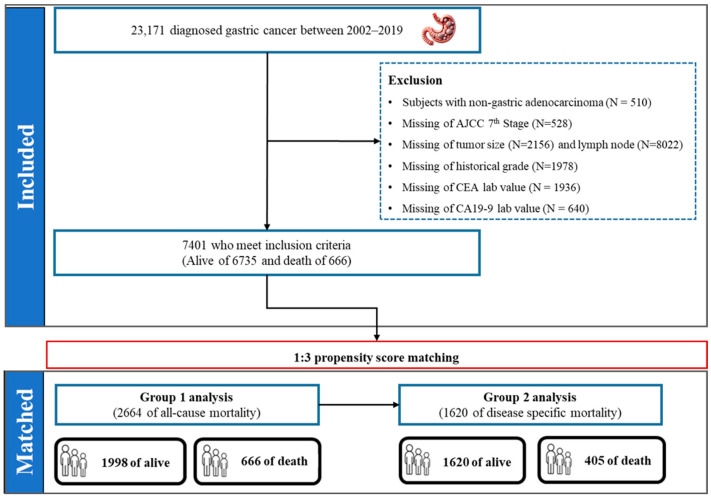
The scheme of patient selection. Total of 23,717 patients diagnosed for the first time between 2002 and 2019. All investigated 6907 patients were divided into two groups for analysis after 1:3 propensity score matching. Group 1: all-cause mortality, and Group 2: disease-specific mortality.

This retrospective study analyzed data from patients diagnosed with GC for the first time between 2002 and 2019 that were included at baseline (Figure 2). All GC patients were defined from C16.1 to 16.9 as Korean certification by Statistics Korea based on the ICD 10th revision (ICD-10). In addition, for all GC types, gastric adenocarcinoma is the most common form of stomach cancer. Initially, total of 23,171 patients were screened for eligibility. We excluded 15,770 patients based on the following criteria: non-gastric adenocarcinoma (N = 510), patients with missing information on AJCC 7th (N = 528), tumor size (N = 2156), lymph node (N = 8022), historical grade (N = 1978), CEA value (N = 1936) and CA19-9 lab value (N = 640). After these exclusions, 7401 patients with a first diagnosis of GC remained, with 666 deaths and 6735 survivors. We analyzed the data for two type groups of disease-specific causes and all-cause. One group included all-cause death for GC patients, while the other included patients for only cause of death by GC.

#### 2.1.3. Definition of Variables

All variables were obtained at the time of the first diagnosis, with missing clinical, pathological, or socio-economic data excluded. Furthermore, the information acquired from self-reported questionnaires, such as check-up frequency, alcohol consumption, smoking, and medical history data, were also excluded if they were empty. Data on smoking, physical activity, drinking, check-up repetition, disability, and history of disease were acquired using self-reported questionnaires. Income, residence, and insurance were acquired from Korean statistics for each patient. Lymph node counts were categorized into four groups according to the following criteria: no regional lymph nodes and metastasis to regional lymph nodes. The AJCC7 tumor stage was classified from I to IV. The primary tumor location was classified into five regions: the fundus, body, antrum, pylorus, and others. Body mass index (BMI, kg/m^2^) was categorized to reflect different levels of obesity: normal (23.0–24.9), overweight (25.0–29.9), and obesity (≥30.0). The type of insurance (national aid, self-employed insured, and employee insured) and household income level were classified into deciles and subsequently categorized into four groups: low (0–2), middle (3–5), middle high (6–8), high (9–10). Residence was designated as either metropolitan or other regions. Disability status was classified into three levels: none, mild, or severe.

### 2.2. Methods

#### 2.2.1. Study Outcomes and Approval

The primary outcome is the prediction of mortality for patients with GC and identifying risk variables utilizing integrated data of clinical, pathological, and social features. To demonstrate risk variables that affect patient mortality prediction, we conducted statistics and ML analysis. In addition, we performed detailed analyses on two groups: disease-specific causes (GC) and all-cause. The Institutional Review Board of the Kangwon National University Hospital approved this study (approval No. KNUH2024-01-005) and waived the requirement for informed consent because anonymized data were used. This study complied with the Declaration of Helsinki and Strengthening the Reporting of Observational Studies in Epidemiology (STROBE) reporting guidelines [50,51].

#### 2.2.2. Data Pre-Processing

Raw data can significantly impact the ML performance of optimized classifiers. Thus, it means that raw data pre-processing is effective in ML algorithms for mortality classification for highlighting high performance. The dataset used in this study comprised information of clinical and laboratory, questionnaire, and lifestyle information by patient subjective and socio-economic data. A few outliers—resulting from incorrect entries by medical staff and research coordinator during the data collection process were removed. All missing values for each feature were removed for using pure raw data. Min–max normalization was conducted on all input variables to prevent issues arising from differences in data scale, enhancing model performance. This normalization step was crucial, as features with larger scales could disproportionately influence the machine learning model compared to those with smaller values [52].

#### 2.2.3. Machine Learning Models

Five machine learning (ML) models were trained to predict mortality: Random Forest (RF) [53], Gradient Boosting Machine (GBM) [54], Extreme Gradient Boosting (XGB) [55], Light Gradient Boosting Machine (LGB) [56], and Categorical Boosting (CatBoost) [57]. RF improves accuracy and robustness through an ensemble of decision trees with bootstrap sampling. GBM enhances predictive performance by iteratively training decision trees to correct previous errors. XGB optimizes gradient boosting using parallel processing and bucket-based data discretization, while LGB increases efficiency with gradient-based sampling and feature bundling. CatBoost handles categorical variables efficiently with minimal preprocessing, employing ordered boosting to mitigate overfitting and target leakage. These algorithms were trained for mortality prediction across two groups based on cause of death (all-cause and disease-specific). All programming was conducted in Python (version 3.8.10) with model construction using Scikit-learn (version 1.2.0). Hyperparameter tuning was performed using Optuna (version 3.3.0), a Bayesian optimization framework for hyperparameter selection [58].

#### 2.2.4. Model Construction and Interpretation

The dataset was split into training (80%) and testing (20%) subsets, with 20% of the training set allocated for validation. Stratified 5-fold cross-validation (CV) was applied to prevent label distortions and ensure model stability. Model performance was evaluated using 1000 bootstrap samples with replacement. Metrics such as the area under the receiver operating characteristic curve (AUC-ROC), Brier score, and the mean with 95% confidence intervals (CIs) were calculated. Performance evaluation included accuracy [59], precision, recall, F1-score, specificity, and AUC-ROC [60,61]. Variable contributions to model performance were analyzed using Shapley Additive Explanation (SHAP) values, providing insights into feature importance and model interpretability [62,63].

#### 2.2.5. Statistical Analysis

To improve robustness and ensure balanced baseline covariates, we conducted a 1:3 nearest-neighbor propensity score matching (PSM) analysis. The quality of the match was evaluated through the standardized mean difference (SMD), with an SMD < 0.1 indicating a negligible difference between the groups (Table S1) [64]. Patients characteristics are expressed as means with standard deviation or numbers (%). An independent *t*-test was performed to assess the differences in the mean between the alive and death groups for continuous variables. Categorical variables are compared between two groups using Chi-squared (χ^2^) tests. In addition, the Cox proportional hazards regression model was analyzed after verifying the proportional hazards assumption test. We assessed the hazard ratio (HR) of all variables using univariate and multivariate Cox regression analysis. All statistical analyses were conducted in R (version 3.8.1). The analysis yielded significant *p*-values for each variable, with all statistical tests conducted at a significance level of *p* < 0.001. All analyses were performed using R software (version 3.6.3).

## 3. Results

### 3.1. Patient Characteristics

The characteristics of individual patients are presented in Table 1. Patient characteristics before PSM are shown in Table S2. We also presented patient characteristics over the ten years, conducting four separate analyses for all-cause and GC-specific mortality groups on whether to conduct PSM (Tables S3–S6). The male deaths predominated in both groups, with 506 (76.0%) in the all-cause group and 291 (71.9%) in the disease-specific group. Significant predictors of mortality included smoking, drinking, CEA levels, tumor size, lymph node count, AJCC7 stage (*p* < 0.001), and physical activity (*p* < 0.05). Notably, current smokers in the all-cause group had a 183 (27.5%) mortality rate, which increased with frequent alcohol consumption. In the disease-specific group, their 102 (25.2%) mortality rate also rose with drinking over three times per week. Furthermore, average tumor sizes were larger in deceased patients, at 49.2 ± 41.1 in the all-cause group and 60.6 ± 44.7 in the disease-specific group. A higher proportion of deceased patients had ≥15 lymph nodes involved, accounting for 251 (37.7%) of all-cause and 185 (45.7%) of disease-specific mortalities. According to the AJCC7 staging system, a significant number of stage IV patients showed elevated mortality rates, with 191 (28.7%) in the all-cause and 176 (43.5%) in the disease-specific categories.

### 3.2. Cox Proportional Hazard Model

Table 2 presents the risk variables for patient mortality. In the all-cause mortality group, obesity, defined by a BMI of 30 or higher, was identified as a significant risk predictor (HR 1.78, 95% CI 1.03–3.05). Specifically, increased tumor size correlated with a slightly higher risk of death (HR 1.01, 95% CI 1.00–1.01). Lymph node counts were associated with a risk (HR 0.81, 95% CI 0.64–0.99). Cancer progression, particularly stage IV under the AJCC7 system, showed significantly higher mortality risks (HR 3.09, 95% CI, 2.28–4.20), leading to a three-fold increase compared to reference-stage patients. Diabetes also has a high risk (HR 1.45, 95% CI 1.19–1.78). In the disease-specific group, CEA (HR 1.00, 95% CI 1.00–1.01), CA19-9 (HR 1.00, 95% CI 1.00–1.01), tumor size (HR 1.01, 95% CI 1.01–1.01), lymph node counts (HR 0.31, 95% CI 0.20–0.47), and AJCC7 stage (HR 3.99, 95% CI 2.72–5.84) were significant predictors. A history of stroke significantly increased stroke risk (HR 2.36, 95% CI 1.60–3.47; and HR 2.09, 95% CI 1.17–3.74 in both groups). Regular health check-ups, over the past decade, consistently emerged as significant protective increasing health screenings for GC significantly lowered cancer risk (0.02 HR, 95% CI 0.00–0.06 in the all-cause group; 0.01 HR, 95% CI 0.00–0.05 in the disease-specific group). Cancer progression, particularly stage IV under the AJCC7 system, showed significantly higher mortality risks (HR 3.99, 95% CI, 2.72–5.84), leading to a four-fold increase compared to reference-stage patients.

### 3.3. Machine Learning Model Performance and Evaluation

Six ML algorithms were used to construct predictive models for GC mortality. The predictive classification results for both groups of six ML algorithms are presented in Table 3.

**Figure 3:**
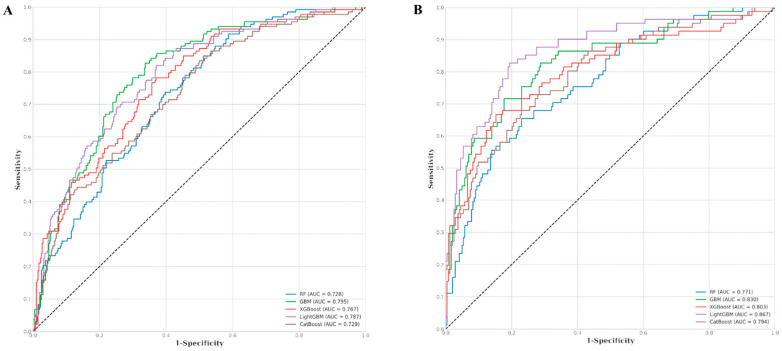
AUROC of model performance. (**A**) All-cause mortality shows best performance in GBM (AUC of 0.795), followed by Light GBM (AUC = 0.787), while the Random Forest (RF) model has the lowest performance (AUC = 0.728). (**B**) Disease-specific mortality shows best performance in LGB (AUC of 0.867), followed by GBM (AUC of 0.830) and XGB (AUC of 0.803). The RF model shows the lowest performance AUC of 0.771. Abbreviation: AUROC, Area Under the Receiver Operating Characteristic Curve; RF, Random Forest; GBM, Gradient Boosting Machine; XGBoost, Extreme Gradient Boosting; Light GBM, Light Gradient Boosting Machine; CAT Boost, Categorical Boosting.

For all-cause mortality, GBM showed the best performance, with a predictive classification AUROC of 0.795 with an accuracy of 0.782, a precision of 0.655, and a specificity of 0.953 (Table 3). The other models had the following AUROC values: LGB, 0.787; XGB, 0.767; CATBoost, 0.729; RF, 0.728 (Figure 3A).

For disease-specific mortality, LGB showed the best performance in predictive classification with an AUROC of 0.867 with an accuracy of 0.849, a precision of 0.796, a specificity of 0.955, an F1 score of 0.637, and an AUROC of 0.867. The other models had the following AUROCs: GBM, 0.830; XGB, 0.803; CAT Boost, 0.794; and RF, 0.771 (Figure 3B).

### 3.4. Machine Learning Interpretation

We demonstrate high-effect variables for GC mortality by SHAP, one of the explainable AI (XAI) methods (Figures S1 and S2). The SHAP values indicate the extent to which each feature contributed to the model prediction, with positive values indicating an increased risk of death and negative values indicating a decreased risk of death. In addition to highlighting their contribution to predictions, SHAP values provide insights into the biological and clinical significance of each feature in relation to GC progression. They help interpret the influence and importance of each feature in the model’s decision-making process. In SHAP interpretation summary figures, the *x*-axis represents the SHAP value, which indicates the effect of a feature on the prediction outcome, and the *y*-axis lists the features. For instance, the AJCC7 stage, which had the highest SHAP values, reflects the extent of tumor progression, with advanced stages being strongly associated with increased mortality due to greater invasiveness and metastatic potential. Tumor size, another high-impact feature, directly correlates with the tumor burden and the likelihood of systemic spread, which aligns with its role as a major prognostic factor. Similarly, elevated levels of CEA and CA19-9, both well-known tumor markers, suggest aggressive cancer behavior and poor survival outcomes.

The plot could provide an information-dense summary of the effects of the top features of a dataset on the model results. For each instance, the explanation is represented by a single dot for each feature flow. The distribution of points on the plot for each variable across the SHAP value axis indicates the degree of the impact of this feature on the output of the model. Each point represents an individual data point in the dataset.

**Figure 4:**
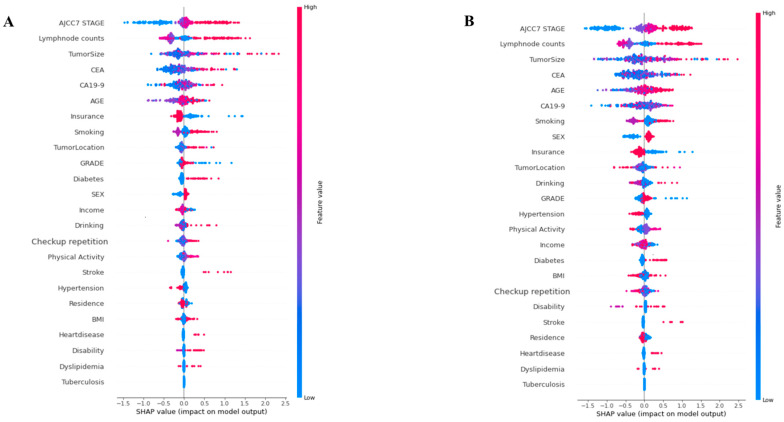
Variables interpretation by SHAP. (**A**) Gradient boosting machine learning model for all-cause mortality. The plot illustrates the contribution of features that affect the prediction of mortality, such as tumor size, AJCC7 stage, CEA, smoking, etc. (**B**) Light gradient boosting machine learning model for disease-specific mortality. The plot illustrates the contribution of features that affect the prediction of mortality, such as AJCC7 stage, lymph node counts, tumor size, CEA, CA19-9, smoking, etc. Abbreviation: BMI, body mass index; CEA, carcinoembryonic antigen; CA19-9, CA 19-9 antigen; AJCC7 STAGE, AJCC Cancer Staging (7th edition); GRADE, Tumor Grade.

Figure 4 shows interpreted risk variables by SHAP for best AUC models in all-cause and disease-specific groups. Among the 10 variables identified by the best model in each group, the common variables were AJCC7 stage, tumor size, lymph node counts, CEA, age, smoking, and CA19-9. These were also reflected in the high-risk variables identified using the Cox proportional hazards model. Specifically, the SHAP results revealed that lifestyle factors, including smoking and drinking, exhibited moderate SHAP values, suggesting their influence on GC outcomes. Smoking is known to promote inflammation, a key factor in tumor progression, while alcohol consumption may weaken immune defenses and exacerbate cancer progression. In detail, in the all-cause mortality group, the factors that increased the risk of death were tumor size, AJCC7 stage, and CEA. AJCC7 stage, positive lymph node count, and tumor size were the most influential variables in the disease-specific mortality group. Notably, the SHAP results align closely with the known clinical pathways of GC progression. For instance, higher lymph node counts signify increased metastatic spread, which is strongly associated with worse survival outcomes. In addition, lifestyle factors such as smoking, drinking, checkup repetition, and physical activity demonstrated a moderate influence. In disease-specific groups, the AJCC7 stage, lymph node counts, tumor size, CEA, and CA19-9 were identified as having a high impact. However, insurance, smoking, drinking, and diabetes were found to have a moderate influence.

The interpretation by SHAP analysis suggested that certain high-impact variables significantly contribute to the model’s predictions, indicating their influence on patient outcomes. Specifically, these findings suggest a positive correlation between these variables and survival rates, implying that an increase in their frequency or grade may positively affect survival outcomes. These findings not only elucidate the model’s prediction patterns but also reinforce the importance of these features in understanding GC biology and progression.

## 4. Discussion

We used nationwide cancer cohort data from the K-CURE, a large clinical registry of 23,717 GC patients. This study is the first to use K-CURE and GC data from the K-CURE dataset to investigate GC patient mortality, identify risk factors, and assess mortality influences. Collected exclusively from the Korean population, this dataset is crucial for ethnic studies, reflecting demographic-specific characteristics such as socioeconomic factors (medical insurance type, income, residential area) and general health screening.

Our research could help improve risk factors in daily life before cancer diagnosis and during treatment by identifying risk factors in GC patients. Through the results, the patient’s quality of life might improve and provide information to effectively manage and monitor risk factors beyond pathology and clinical information after treatment in the future. Five ML models were evaluated for two groups of all-cause and disease-specific mortality. In particular, we verified the robustness of the model performance, to cover a small sample size, we conducted an assessment using 1000 iteration bootstrap sampling from the entire test dataset. GBM and LGB achieved the best discrimination AUC performance and demonstrated stable accuracy by low Brier scores in each group (Table 3). Furthermore, predictive classification performance stayed robust, underscoring the impact of clinical variables and lifestyle factors on mortality prediction. The optimized model demonstrated favorable discrimination for each group, and as a result, the models’ explainability was performed in this optimal model to improve the models’ trust and transparency. These models highlight the importance of controlling variables like smoking, drinking, body weight, physical activity, and clinical and pathological data.

In this study, we suggested several strengths. First, our study utilized integrated data, including lifestyle, clinical, and socioeconomic information, to predict mortality in GB patients. Mortality is influenced not only by treatment procedures in hospitals but also by lifestyle factors. Annual health check-ups prior to cancer diagnosis, active exercise, smoking, drinking and body fat, and diabetes management significantly reduce cancer risk. Guo’s research highlights that lifestyle behaviors such as smoking, alcohol consumption, and diet significantly increase the risk and mortality of GC [65]. Jiang’s study identified key factors for GC diagnosis, including age, family history, smoking, drinking, fruit and vegetable intake, H. pylori infection, and PGII levels [66]. Similarly, Ko developed a mortality prediction model for GC based on body morphometry, nutritional status, surgical outcomes, and clinicopathological data [67]. While prior studies have primarily focused on either lifestyle-related risk factors for disease onset or clinical data for mortality prediction, our study integrates both lifestyle and clinical information to predict GC mortality more comprehensively. Second, the risk factors identified in our study are consistent with previous research findings. In our study, variables that affect all-cause mortality include the AJCC7 stage, lymph node counts, tumor size, CEA, and CA19-9 [68,69,70,71,72]. In addition, most of the risk factors in disease-specific mortality prediction results are similar to all-cause mortality prediction with high importance of smoking [65,73]. These results are similar to those presented in previous studies and can provide reliability of our study results. Third, our study focuses on Asian datasets, helping to bridge the gap in cancer research, which is often centered on Western populations. The dataset, Korea’s largest clinical cancer database, was developed through collaborations among oncology, healthcare, and data science experts from leading South Korean hospitals. Fourth, our ML models outperformed existing tools for several reasons. Unlike logistic regression, advanced ML techniques excel at modeling nonlinear and complex relationships between variables [74,75], which is crucial in healthcare, where outcomes are influenced by diverse demographic and clinical factors. Among the models, GBM and LGB demonstrated superior performance, offering benefits such as reduced overfitting risks, faster computation, and high precision [54,56]. Moreover, compared to many existing GC studies that applied ML, our results provide high accuracy with other metrics for specific investigation of the cause of death, as presented in Table 4 [7,69,76,77,78,79]. Fifth, the risk variables identified in our model were consistent across the statistical model and the XAI. Importantly, these factors emerged from interactions among demographics and clinical and pathological data. Specifically, addressing systemic and structural inequities related to health’s social determinants, such as economic stability, medical aid, and national health insurance, is crucial for reducing GC mortality.

Our study also has some limitations. First, our models were developed using the K-CURE, a South Korean voluntary registry. Future studies must encompass diverse ethnicities to validate the findings globally and ensure their generalizability. Integrating data from multiple institutions faces challenges due to reporting delays across organizations, affecting data accuracy and consistency. Employing numerous variables to represent varied data sources could diminish the generalizability necessary for ML applications, potentially limiting longitudinal research methods. Second, data from multicenter hospitals varied due to the discretionary selection of data items. In real-world scenarios where each institution employs a different healthcare information system, variations in collectible data are inevitable. To minimize this disparity, all data could be extracted in a structured format. Third, this study, initiated with over 20,000 patients, utilized broad selection criteria to minimize selection bias. However, more detailed information is not in the cohort. Although all GC patients investigated in our study underwent chemotherapy and were considered to define GC grade considering poorly differentiated and signet ring cells, more information is lacking. We could not investigate for detailed information related to GC, such as what nutrients the patient consumed or information about Helicobacter pylori was not available. In addition, certain parameters were challenging to identify, including secondary features like invasive surgery and lymphadenectomy in relation to the progression of gastric cancer. Beyond D1 and D2 lymphadenectomy, which are known to significantly influence gastric cancer mortality, procedures such as para-aortic lymphadenectomy may also be necessary [80]. These reasons might lead to limitations in decisions and considerations regarding whether a patient has undertaken an accurate diagnosis and therapeutic process. Moreover, the data structure limited the application of longitudinal research methods, and there was no follow-up data on disease recurrence or progression. Fourth, the study design did not allow for the identification of causal associations. Although a randomized clinical trial is precluded by ethical concerns, the time-exposure study design provides an opportunity to explore potential biological associations. Fifth, it remains challenging to demonstrate the generalizability of the model. In this study, we presented a framework that compares and optimizes multiple ML models using a large cohort dataset collected from multiple medical institutions. While this approach enhances the reliability of model results by utilizing data from patients across various institutions, it does not directly address the generalizability of the model itself. Recently, methodologies such as federated learning have been proposed for leveraging large-scale, multi-institutional datasets. Federated learning involves a collaborative framework where multiple devices or institutions solve machine learning problems by aggregating and transferring knowledge from distributed local datasets [81]. This approach ensures data security while allowing the continuous update of model weights on a central server, ultimately improving the generalizability of the resulting model. However, our study utilized a pre-compiled dataset provided as a single database, which aggregated data from multiple medical institutions. This setup precluded the separation of data by individual institutions during the learning process. Future research incorporating federated learning methodologies, particularly in the context of gastric cancer studies, could further enhance the generalizability of ML models by leveraging in-depth analyses and institution-specific data distributions.

## 5. Conclusions

In conclusion, we demonstrated the predictive classification of GC mortality using the K-CURE dataset as a large cohort in South Korea. By utilizing a real-world data-integrated clinical and socio-economic database, we demonstrate that incorporating variables beyond clinical factors offers a more accurate representation of real-world outcomes. We developed and validated five ML models and suggested optimized ML models, such as LGB and GBM, that showed strong predictive performance for disease-specific and all-cause mortality. Furthermore, through XAI, we identified key risk factors, including AJCC stage, tumor size, lymph node count, and lifestyle factors like smoking, drinking, and diabetes. This research advances personalized treatment strategies for GC in the Korean population, emphasizing the need for demographic-specific data in predictive models.

## Figures and Tables

**Table 1:** Patients characteristics.

	All Cause Group				Disease Specific Group			
	Total (N = 2664)	Alive (N = 1998)	Death (N = 666)	*p*	Total (N = 1620)	Alive (N = 1215)	Death (N = 405)	*p*
**Age**	67.4 ± 11.0	67.4 ± 11.0	67.4 ± 11.1	0.932	65.5 ± 12.0	65.5 ± 12.0	65.6 ± 12.1	0.947
**SEX (Male)**	742 (27.9)	582 (29.1)	160 (24.0)	<0.05	502 (31.0)	388 (31.9)	144 (35.6)	0.172
**BMI**								0.153
Normal	1848 (69.4)	1381 (69.1)	467 (70.1)		1098 (67.8)	819 (67.4)	279 (68.9)	
Overweight	784 (29.4)	599 (30.0)	185 (27.8)		500 (30.9)	383 (31.5)	117 (28.9)	
Obesity	32 (1.2)	18 (0.9)	14 (2.1)		22 (1.4)	13 (1.1)	9 (2.2)	
**Smoking**				<0.001				<0.001
Never	1276 (47.9)	959 (48.0)	317 (47.6)		801 (49.4)	596 (49.1)	205 (50.6)	
Former	821 (30.8)	655 (32.8)	166 (24.9)		506 (31.2)	408 (33.6)	98 (24.2)	
Current	567 (21.3)	384 (19.2)	183 (27.5)		313 (19.3)	211 (17.4)	102 (25.2)	
**Drinking (in a week)**				<0.001				<0.05
<1	1536 (57.7)	1144 (57.3)	392 (58.9)		934 (57.7)	692 (57.0)	242 (59.8)	
1–3	858 (32.2)	667 (33.4)	191 (28.7)		549 (33.9)	430 (35.4)	119 (29.4)	
4–6	192 (7.2)	144 (7.2)	48 (7.2)		99 (6.1)	74 (6.1)	25 (6.2)	
7	78 (2.9)	43 (2.2)	35 (5.3)		38 (2.3)	19 (1.6)	19 (4.7)	
**Tumor Location**				<0.001				<0.001
Fundus	10 (0.4)	6 (0.3)	4 (0.6)		8 (0.5)	5 (0.4)	3 (0.7)	
Body	1156 (43.4)	863 (43.2)	293 (44.0)		751 (46.4)	567 (46.7)	184 (45.4)	
Antrum	1231 (46.2)	957 (47.9)	274 (41.1)		694 (42.8)	542 (44.6)	152 (37.5)	
Pylorus	35 (1.3)	20 (1.0)	15 (2.3)		18 (1.1)	6 (0.5)	12 (3.0)	
Others	232 (8.7)	152 (7.6)	80 (12.0)		149 (9.2)	95 (7.8)	54 (13.3)	
**CEA**	28 ± 30.0	26 ± 27.0	33 ± 37.2	<0.001	29 ± 34.2	27 ± 31.2	34 ± 41.4	<0.05
**CA19-9**	91 ± 66.8	90 ± 66.1	95 ± 68.9	0.104	93 ± 68.0	91 ± 66.6	97 ± 72.0	0.1
**Tumor Size**	41 ± 31.0	38 ± 26.2	49 ± 41.1	<0.001	48 ± 33.5	44 ± 27.7	61 ± 44.7	<0.001
**Lymph Node Count**				<0.001				<0.001
0	1039 (39.0)	806 (40.3)	233 (35.0)		393 (24.3)	316 (26.0)	77 (19.0)	
1–6	521 (19.6)	407 (20.4)	114 (17.1)		417 (25.7)	331 (27.2)	86 (21.2)	
7–15	388 (14.6)	320 (16.0)	68 (10.2)		347 (21.4)	290 (23.9)	57 (14.1)	
≥16	716 (26.9)	465 (23.3)	251 (37.7)		463 (28.6)	278 (22.9)	185 (45.7)	
**AJCC7 STAGE**				<0.001				<0.001
I	1375 (51.6)	1095 (54.8)	280 (42.0)		502 (31.0)	427 (35.1)	75 (18.5)	
II	368 (13.8)	272 (13.6)	96 (14.4)		277 (17.1)	211 (17.4)	66 (16.3)	
III	495 (18.6)	396 (19.8)	99 (14.9)		445 (27.5)	357 (29.4)	88 (21.7)	
IV	426 (16.0)	235 (11.8)	191 (28.7)		396 (24.4)	220 (18.1)	176 (43.5)	
**GRADE**				<0.05				<0.05
1	374 (14.0)	275 (13.8)	99 (14.9)		110 (6.8)	81 (6.7)	29 (7.2)	
2	1072 (40.2)	842 (42.1)	230 (34.5)		599 (37.0)	478 (39.3)	121 (29.9)	
3	1218 (45.7)	881 (44.1)	337 (50.6)		911 (56.2)	656 (54.0)	255 (63.0)	
**Physical Activity (in a week)**				<0.05				<0.05
<1	1022 (38.4)	768 (38.4)	254 (38.1)		603 (37.2)	449 (37.0)	154 (38.0)	
1–3	1348 (50.6)	1002 (50.2)	346 (52.0)		842 (52.0)	633 (52.1)	209 (51.6)	
4–5	203 (7.6)	156 (7.8)	47 (7.1)		121 (7.5)	90 (7.4)	31 (7.7)	
≥6	91 (3.4)	72 (3.6)	19 (2.9)		54 (3.3)	43 (3.5)	11 (2.7)	
**Check-up Repetition** **(in the past 10 years)**				0.382				0.391
Never	737 (27.7)	551 (27.6)	186 (27.9)		507 (31.3)	378 (31.1)	129 (31.9)	
1–2	1370 (51.4)	1031 (51.6)	339 (50.9)		737 (45.5)	554 (45.6)	183 (45.2)	
3–4	406 (15.2)	297 (14.9)	109 (16.4)		266 (16.4)	195 (16.0)	71 (17.5)	
5–6	129 (4.8)	102 (5.1)	27 (4.1)		89 (5.5)	72 (5.9)	17 (4.2)	
7–8	16 (0.6)	14 (0.7)	2 (0.3)		15 (0.9)	13 (1.1)	2 (0.5)	
9–10	6 (0.2)	3 (0.2)	3 (0.5)		6 (0.4)	3 (0.2)	3 (0.7)	
**Insurance**				<0.001				<0.05
Medical aids	31 (1.2)	14 (0.7)	17 (2.6)		15 (0.9)	6 (0.5)	9 (2.2)	
Self-employed insured	682 (25.6)	500 (25.0)	182 (27.3)		437 (27.0)	317 (26.1)	120 (29.6)	
Employee insured	1951 (73.2)	1484 (74.3)	467 (70.1)		1168 (72.1)	892 (73.4)	276 (68.1)	
**Residence (Urban)**	1113 (41.8)	842 (42.1)	271 (40.7)	0.891	710 (43.8)	532 (43.8)	178 (44.0)	0.562
**Income**				0.07				0.333
Low	385 (14.5)	268 (13.4)	117 (17.6)		250 (15.4)	176 (14.5)	74 (18.3)	
Middle low	616 (23.1)	466 (23.3)	150 (22.5)		374 (23.1)	282 (23.2)	92 (22.7)	
Middle high	788 (29.6)	597 (29.9)	191 (28.7)		484 (29.9)	367 (30.2)	117 (28.9)	
High	875 (32.8)	667 (33.4)	208 (31.2)		512 (31.6)	390 (32.1)	122 (30.1)	
**Disability**				<0.05				0.087
No	2388 (89.6)	1804 (90.3)	584 (87.7)		1491 (92.0)	1121 (92.3)	370 (91.4)	
Severe	93 (3.5)	73 (3.7)	20 (3.0)		56 (3.5)	46 (3.8)	10 (2.5)	
Mild	183 (6.9)	121 (6.1)	62 (9.3)		73 (4.5)	48 (4.0)	25 (6.2)	
**Stroke (Yes)**	125 (4.7)	80 (4.0)	45 (6.8)	<0.05	72 (4.4)	53 (4.4)	19 (4.7)	0.064
**Heart Disease (Yes)**	125 (4.7)	80 (4.0)	45 (6.8)	<0.05	72 (4.4)	53 (4.4)	19 (4.7)	0.889
**Hypertension (Yes)**	936 (35.1)	695 (34.8)	241 (36.2)	0.542	524 (32.3)	389 (32.0)	135 (33.3)	0.668
**Diabetes (Yes)**	350 (13.1)	229 (11.5)	121 (18.2)	<0.001	183 (11.3)	122 (10.0)	61 (15.1)	<0.05
**Tuberculosis (Yes)**	27 (1.0)	19 (1.0)	8 (1.2)	0.738	11 (0.7)	8 (0.7)	3 (0.7)	1
**Dyslipidemia (Yes)**	104 (3.9)	79 (4.0)	25 (3.8)	0.908	58 (3.6)	43 (3.5)	15 (3.7)	1

The reference categories are defined as follows: SEX: male; Residence: urban; Stroke: presence of dyslipidemia. All *p*-values are derived from two-group analyses comparing Alive and Death outcomes. All variables presented as means with standard deviation for continuous factors and numbers (%) for categorical factors. The differences among groups were evaluated using two-tailed *t*-test for continuous variables and χ^2^ test for categorical variables. Abbreviation: BMI, body mass index; CEA, carcinoembryonic antigen; CA19-9, CA 19-9 Antigen; AJCC7 STAGE, AJCC Cancer Staging (7th edition); GRADE, Tumor Grade.

**Table 2:** Results of Cox proportional hazard.

	All Cause Group				Disease Specific Group			
	Univariate		Multivariate		Univariate		Multivariate	
	HR (95% CI)	*p*	HR (95% CI)	*p*	HR (95% CI)	*p*	HR (95% CI)	*p*
**Age**	1.00 (0.99–1.01)	0.567			1.00 (0.99–1.00)	0.321		
**SEX**								
Male	1.24 (1.04–14.80)	<0.05	1.53 (1.22–1.92)	<0.001	1.17 (0.94–1.45)	0.156		
**BMI**								
Overweight	0.90 (0.76–1.07)	0.245	0.89 (0.74–1.06)	0.177	0.90 (0.73–1.12)	0.358		
Obesity	1.89 (1.11–1.23)	<0.05	1.78 (1.03–3.05)	<0.05	1.87 (0.96–3.63)	0.065		
**Smoking**								
Former	0.82 (0.68–0.99)	<0.05	0.65 (0.52–0.82)	<0.001	0.75 (0.59–0.96)	<0.05	0.73 (0.56–0.96)	<0.05
Current	1.47 (1.23–1.76)	<0.001	1.14 (0.91–1.42)	0.253	1.59 (1.26–2.02)	<0.001	1.38 (1.06–1.79)	<0.05
**Drinking (days/week)**								
1–3	0.90 (0.76–1.07)	0.226	0.87 (0.71–1.05)	0.146	0.87 (0.70–1.08)	0.209	0.86 (0.67–1.10)	0.254
4–6	1.10 (0.82–1.49)	0.525	1.09 (0.79–1.49)	0.607	1.12 (0.74–1.69)	0.587	1.26 (0.82–1.96)	0.283
7	2.30 (1.62–2.25)	<0.001	2.00 (1.39–2.88)	<0.001	2.23 (1.40–3.56)	<0.05	2.06 (1.26–3.38)	<0.001
**Tumor Location**								
Body	0.73 (0.27–1.95)	0.529			0.80 (0.25–2.50)	0.7		
Antrum	0.65 (0.24–1.76)	0.4			0.72 (0.23–2.27)	0.557		
Pylorus	1.21 (0.40–3.66)	0.733			2.37 (0.67–8.41)	0.189		
Others	1.00 (0.37–2.72)	0.996			1.18 (0.37–3.79)	775		
**CEA**	1.00 (1.00–1.01)	<0.001	1.00 (1.00–1.01)	<0.05	1.00 (1.00–1.01)	<0.05	1.00 (1.00–1.00)	0.254
**CA19-9**	1.00 (1.00–1.00)	0.055			1.00 (1.00–1.00)	<0.05	1.00 (1.00–1.00)	<0.05
**Tumor Size**	1.01 (1.01–1.01)	<0.001	1.01 (1.00–1.01)	<0.001	1.01 (1.01–1.01)	<0.001	1.01 (1.01–1.01)	<0.001
**Lymph Node Count**								
1–6	0.75 (0.57–0.10)	<0.05	0.35 (0.25–0.50)	<0.001	0.88 (0.62–1.24)	0.453	0.53 (0.36–0.78)	<0.001
7–15	0.99 (0.79–1.24)	0.962	0.59 (0.44–0.78)	<0.001	1.21 (0.89–1.65)	0.217	0.31 (0.20–0.47)	<0.001
≥16	1.39 (1.16–1.66)	<0.001	0.81 (0.64–1.01)	<0.05	2.04 (1.56–2.66)	<0.001	0.85 (0.60–1.21)	0.386
**AJCC7 STAGE**								
II	1.06 (0.84–1.33)	0.645	1.79 (1.35–2.37)	<0.001	1.57 (1.15–2.13)	<0.05	2.22 (1.54–3.19)	<0.001
III	1.34 (1.07–1.69)	<0.05	1.80 (1.29–2.51)	<0.05	1.83 (1.31–2.55)	<0.001	2.40 (1.61–3.59)	<0.001
IV	2.27 (1.88–2.73)	<0.001	3.09 (2.28–4.20)	<0.001	3.39 (2.58–4.44)	<0.001	3.99 (2.72–5.84)	<0.001
**GRADE**								
2	0.73 (0.57–0.92)	<0.05	0.68 (0.53–0.88)	<0.05	0.68 (0.46–1.03)	0.067		
3	1.02 (0.81–1.27)	0.89	0.83 (0.64–1.08)	0.157	1.09 (0.74–1.60)	0.669		
**Physical Activity (days/week)**								
1–3	1.08 (0.92–1.27)	0.37			1.07 (0.87–1.32)	0.506		
4–5	0.95 (0.69–1.29)	0.727			1.04 (0.71–1.53)	0.837		
≥6	0.93 (0.58–1.48)	0.758			0.97 (0.53–0.18)	0.929		
**Check-up Repetition**								
1–2	0.56 (0.46–0.68)	<0.001	0.60 (0.49–0.74)	<0.001	0.53 (0.41–0.68)	<0.001	0.54 (0.41–0.70)	<0.001
3–4	0.11 (0.08–0.16)	<0.001	0.12 (0.09–0.17)	<0.001	0.10 (0.06–0.14)	<0.001	0.10 (0.06–0.15)	<0.001
5–6	0.04 (0.02–0.06)	<0.001	0.04 (0.02–0.06)	<0.001	0.03 (0.01–0.05)	<0.001	0.02 (0.01–0.05)	<0.001
7–8	0.02 (0.00–0.07)	<0.001	0.01 (0.00–0.05)	<0.001	0.01 (0.00–0.06)	<0.001	0.00 (0.00–0.04)	<0.001
9–10	0.04 (0.01–0.13)	<0.001	0.02 (0.00–0.06)	<0.001	0.03 (0.01–0.11)	<0.001	0.01 (0.00–0.05)	<0.001
**Insurance**								
Self-employed insured	0.43 (0.26–0.71)	<0.05	0.37 (0.22–0.64)	<0.001	0.52 (0.26–1.02)	0.057	0.38 (0.18–0.76)	<0.05
Employee insured	0.34 (0.21–0.56)	<0.001	0.36 (0.21–0.61)	<0.001	0.40 (0.20–0.77)	<0.05	0.40 (0.20–0.79)	<0.05
**Residence**								
Rural	1.02 (0.88–1.19)	0.771			0.90 (0.74–1.10)	<0.05		
**Income**								
Middle low	0.79 (0.62–1.01)	0.058	0.86 (0.67–1.11)	0.248	0.84 (0.62–1.14)	0.264		
Middle high	0.78 (0.62–0.99)	<0.05	0.78 (0.61–1.00)	0.053	0.80 (0.60–1.07)	0.135		
High	0.77 (0.61–0.96)	<0.05	0.80 (0.62–1.02)	0.074	0.80 (0.60–1.07)	0.128		
**Disability**								
Severe	0.82 (0.53–1.28)	0.389	0.73 (0.46–1.16)	0.182	0.63 (0.34–1.19)	0.155		
Mild	1.49 (1.14–1.93)	<0.05	1.19 (0.90–1.56)	0.218	1.45 (0.97–2.17)	0.072		
**Stroke**								
Yes	2.13 (1.47–3.10)	<0.001	2.36 (1.60–3.47)	<0.001	2.22 (1.28–3.87)	<0.05	2.09 (1.17–3.74)	<0.05
**Heart Disease**								
Yes	1.43 (1.05–1.93)	<0.05	1.47 (1.08–2.01)	<0.05	0.96 (0.61–1.53)	0.873		
**Hypertension**								
Yes	1.06 (0.90–1.24)	0.492			1.03 (0.84–1.27)	0.766		
**Diabetes**								
Yes	1.53 (1.26–1.86)	<0.001	1.45 (1.19–1.78)	<0.001	1.45 (1.11–1.91)	<0.05	1.31 (0.99–1.73)	0.057
**Tuberculosis**								
Yes	1.53 (1.26–1.86)	0.215			1.63 (1.52–5.07)	0.402		
**Dyslipidemia**								
Yes	0.73 (0.49–1.09)	0.124			0.82 (0.49–1.37)	0.444		

Univariate and multivariate Cox regression analysis. Multivariate analysis for factors having statistical significance (*p* < 0.05). The reference categories for all individual risk ratio analyses are defined as follows: SEX: female; BMI: normal; Smoking: never; Drinking: none; Tumor location: fundus; Lymph node count: none; AJCC7 STAGE: grade I; GRADE: 1; Physical activity: none; Check-up repetition: none; Insurance: medical aid; Residence: rural; Income: low; Disability to Dyslipidemia: No. Abbreviation: BMI, body mass index; CEA, carcinoembryonic antigen; CA19-9, CA 19-9 Antigen; AJCC7 STAGE, AJCC Cancer Staging (7th edition); GRADE, Tumor Grade.

**Table 3:** Machine learning performance.

		AUROC	Accuracy	Brier Score	Precision	F1 Score	Specificity
**All cause**	**RF**	0.738 (0.68, 0.77)	0.679 (0.64, 0.71)	0.221 (0.21, 0.22)	0.397	0.457	0.725
	**GBM**	0.795 (0.75, 0.83)	0.782 (0.74, 0.81)	0.154 (0.13, 0.17)	0.655	0.383	0.953
	**XGB**	0.767 (0.72, 0.81)	0.792 (0.75, 0.82)	0.155 (0.13, 0.17)	0.69	0.419	0.955
	**LGB**	0.787 (0.74, 0.82)	0.781 (0.74, 0.81)	0.153 (0.13, 0.17)	0.582	0.494	0.898
	**CAT**	0.729 (0.67, 0.77)	0.781 (0.74, 0.81)	0.177 (0.16, 0.19)	0.589	0.475	0.908
**Disease specific**	**RF**	0.771 (0.71, 0.82)	0.747 (0.70, 0.79)	0.202 (0.19, 0.21)	0.495	0.539	0.798
	**GBM**	0.830 (0.77, 0.88)	0.821 (0.77, 0.86)	0.133 (0.10, 0.16)	0.795	0.517	0.967
	**XGB**	0.803 (0.74, 0.85)	0.809 (0.76, 0.84)	0.136 (0.11, 0.16)	0.744	0.483	0.959
	**LGB**	0.867 (0.81, 0.91)	0.849 (0.80, 0.89)	0.115 (0.09, 0.14)	0.796	0.637	0.955
	**CAT**	0.794 (0.73, 0.84)	0.799 (0.75, 0.84)	0.147 (0.13, 0.16)	0.648	0.519	0.922

Model performance for each group. GBM shows the highest AUC in all-cause mortality and LGB presents the highest AUC. Abbreviation: AUROC, Area Under ROC Curve; CI, Confidence Interval; RF, Random Forest; GBM, Gradient Boosting Machine; XGB, eXtreme Gradient Boosting; LGB, Light Gradient Boosting Machine; CAT, Categorical Boost.

**Table 4:** Systematic review of model performance and variable comparison across studies.

Study	Patients	Models	Observation	AUC	Accuracy	Sensitivity	Specificity	F1 Score	Risky Variables
Afrash, M.R, et al. [7].	974	SVM, XGBoost, hist gradient boosting		0.894	0.891	0.894	0.872	0.908	Tumor stage, tumor site, tumor size, and history of other cancers.
Liu, et al. [70].	2546	RF, cv-Enet, and glm-boost, ensemble		0.716	NA	0.074	0.984	NA	Age, ASA score, tumor size, preoperative serum albumin level, ECOG, neoadjuvant treatment.
Pera, et al. [71].	3182	cv-Enet, glmboost, RF, ensemble		0.844	NA	NA	NA	NA	Sex, age, ECOG performance status, ASA grade, hemoglobin levels, albumin levels, myocardial infarction.
SenthilKumar, et al. [72].	39,108	Ensemble, XGB		0.77	0.85	0.44	0.89	0.34	Age, duration between diagnosis and start of treatment, lymph node counts, neoadjuvant radiation therapy, neoadjuvant chemotherapy, tumor size.
Altinsoy, et al. [73].	34,417	Logistic regression, naïve bayes, multilayer perceptron, bagging, j48, hybrid model		0.847	0.87	NA	NA	0.87	Age, race, sex, tumor primary site, grade, histology, T Stage, N Stage, M Stage, tumor size, follow-up time.
Dal Cero, et al. [74].	1015	KNN, XGB, RF		0.808	0.836	0.928	0.676	0.415	Age, lymph node counts, tumor peripheral nerve invasion (PNI), tumor size, CEA, CA125.
**Our study**	**2664**	**RF, GBM, XGB, LGB, CAT**	**All cause**	**0.787**	**0.781**		**0.898**	**0.494**	**AJCC stage, lymph node counts, tumor size, CEA, CA19-9, age, national insurance.**
			**Disease specific**	**0.867**	**0.849**		**0.955**	**0.637**	**AJCC stage, lymph node counts, tumor size, CEA, CA19-9, age, smoking.**

Abbreviation: AUROC, Area Under ROC Curve; RF, Random Forest; GBM, Gradient Boosting Machine; XGB, eXtreme Gradient Boosting; LGB, Light Gradient Boosting Machine; CAT, Categorical Boost; CEA, carcinoembryonic antigen; carbohydrate antigen; AJCC, American Joint Committee on Cancer.

## Data Availability

The data presented in this study are available on request from the corresponding author.

## Supplementary Materials

The following supporting information can be downloaded at: https://www.mdpi.com/article/10.3390/cancers17010030/s1, Figure S1. Variables interpretation by SHAP for all-cause mortality. (A) Random Forest (B) Light Gradient Boosting Machine (C) Extreme Gradient Boosting (D) Categorical Boosting. The common factors by SHAP interpretation revealed that the AJCC 7th stage, lymph node counts, tumor size, CEA levels, and CA19-9 levels had the most significant impact in relation to mortality risk. Abbreviation: BMI, body mass index; CEA, carcinoembryonic antigen; CA19-9, CA 19-9 Antigen; AJCC7 STAGE, AJCC Cancer Staging (7th edition); GRADE, Tumor Grade; Figure S2. Variables interpretation by SHAP for disease-specific mortality. (A) Random Forest (B) Gradient Boosting Machine (C) Extreme Gradient Boosting (D) Categorical Boosting. The common factors by SHAP interpretation revealed that AJCC 7th stage, diabetes, tumor size, lymph node counts, and CEA levels had the most significant impact in relation to mortality risk. Abbreviation: BMI, body mass index; CEA, carcinoembryonic antigen; CA19-9, CA 19-9 Antigen; AJCC7 STAGE, AJCC Cancer Staging (7th edition); GRADE, Tumor Grade; Table S1. Standardized mean differences between before and after propensity score matching. Table S2. Patient characteristics before propensity score matching. Table S3. Patient characteristics before propensity score matching in biennial for all-cause mortality group. Table S4. Patient characteristics after propensity score matching in biennial for all-cause mortality group. Table S5. Patient characteristics before propensity score matching in biennial for disease-specific mortality group. Table S6. Patient characteristics after propensity score matching in biennial for disease-specific mortality group.
