# Supplementary material for: Predictive Mortality and Gastric Cancer Risk Using Clinical and Socio-Economic Data: A Nationwide Multicenter Cohort Study
Source: Cancers (Basel). 2024 Dec 25;17(1):30. doi: 10.3390/cancers17010030 (PMC11718814; doi:10.3390/cancers17010030)
Supplement: Supplementary file 1 [file cancers-17-00030-s001.zip › Supplementary Tables.pdf]

Supplementary Table S1. Standardized mean differences between before and after propensity score matching.

	All cause group		Disease specific group	
	Before PSM	After PSM	Before PSM	After PSM
**AGE**	0.52	0.00	0.33	0.00
**SEX**				
Female	0.13	0.12	0.03	0.08
Male	0.13	0.12	0.03	0.08
**BMI**				
Normal	0.18	0.02	0.15	0.03
Overweight	0.14	0.05	0.12	0.06
Obesity	0.11	0.10	0.10	0.09
**Smoking**				
Never	0.02	0.01	0.08	0.03
Former	0.05	0.17	0.07	0.21
Current	0.03	0.20	0.02	0.19
**Drinking (days/week)**				
<1	0.12	0.03	0.14	0.06
1-3	0.15	0.10	0.13	0.03
4-6	0.02	0.00	0.06	0.00
7	0.08	0.17	0.05	0.18
**Tumor Location**				
Fundus	0.03	0.05	0.04	0.04
Body	0.04	0.02	0.07	0.03
Antrum	0.15	0.14	0.22	0.14
Pylorus	0.08	0.10	0.12	0.19
Others	0.14	0.15	0.17	0.18
**CEA**	0.29	0.23	0.29	0.20
**CA19-9**	0.12	0.07	0.15	0.09
**Tumor Size**	0.65	0.32	0.93	0.44
**Lymph Node Count**				
0	0.42	0.11	0.82	0.17
1-6	0.17	0.08	0.27	0.14
7-15	0.30	0.17	0.41	0.25

≥16	0.16	0.32	0.32	0.50
**AJCC7 STAGE**				
I	0.98	0.26	1.75	0.38
II	0.16	0.02	0.21	0.03
III	0.37	0.13	0.54	0.18
IV	0.80	0.43	1.14	0.57
**GRADE**				
1	0.31	0.03	0.56	0.02
2	0.07	0.16	0.17	0.20
3	0.33	0.13	0.59	0.18
**Physical activity (days/week)**				
<1	0.02	0.01	0.02	0.02
1-3	0.02	0.04	0.01	0.01
4-5	0.07	0.03	0.04	0.01
≥6	0.01	0.04	0.00	0.05
**Check-up repetition**				
Never	0.19	0.01	0.28	0.02
1-2	0.17	0.01	0.06	0.04
3-4	0.13	0.04	0.09	0.04
5-6	0.31	0.05	0.30	0.08
7-8	0.20	0.06	0.18	0.07
9-10	0.11	0.06	0.07	0.07
**Insurance**				
National aids	0.05	0.15	0.03	0.15
Self-employed insured	0.05	0.05	0.00	0.08
Employee insured	0.04	0.09	0.01	0.12
**Residence**				
Urban	0.12	0.03	0.05	0.00
Rural	0.12	0.03	0.05	0.00
**Income**				
Low	0.02	0.12	0.00	0.04
Middle low	0.01	0.02	0.02	0.01
Middle high	0.05	0.03	0.04	0.10
High	0.02	0.05	0.04	0.03

**Disability**					
	No	0.08	0.08	0.04	0.03
	Severe	0.02	0.04	0.02	0.08
	Mild	0.08	0.12	0.03	0.10
**Stroke**					
	No	0.13	0.13	0.07	0.11
	Yes	0.13	0.13	0.07	0.11
**Heart disease**					
	No	0.10	0.12	0.01	0.02
	Yes	0.10	0.12	0.01	0.02
**Hypertension**					
	No	0.06	0.03	0.00	0.03
	Yes	0.06	0.03	0.00	0.03
**Diabetes**					
	No	0.10	0.19	0.01	0.15
	Yes	0.10	0.19	0.01	0.15
**Tuberculosis**					
	No	0.02	0.02	0.06	0.01
	Yes	0.02	0.02	0.06	0.01
**Dyslipidemia**					
	No	0.16	0.01	0.16	0.01
	Yes	0.16	0.01	0.16	0.01

Abbreviation: PSM, Propensity Score Matching; BMI, Body Mass Index; SMD, Standardized Mean Difference; CEA, Carcinoembryonic Antigen; CA19-9, CA 19-9 Antigen; AJCC7 STAGE, AJCC Cancer Staging (7th edition); GRADE, Tumor Grade.

Supplementary Table S2. Patients characteristics before propensity score matching.

	All cause group				Disease specific group			
	Total (N=6,907)	Alive (N=6,241)	Death (N=666)	*P*	Total (N=6,646)	Alive (N=6,241)	Death (N=405)	*P*
**AGE**	62.4 ± 10.6	61.9 ± 10.4	67.4 ± 11.1	<0.001	62.1 ± 10.5	61.9 ± 10.4	65.6 ± 12.1	<0.001
**SEX**				<0.05				0.506
Female	4,893 (70.8)	4,387 (70.3)	506 (76.0)		4,678 (70.4)	4,387 (70.3)	291 (71.9)	
Male	2,014 (29.2)	1,854 (29.7)	160 (24.0)		1,968 (29.6)	1,854 (29.7)	114 (28.1)	
**BMI**				<0.001				<0.05
Normal	4,320 (62.5)	3,853 (61.7)	467 (70.1)		4,132 (62.2)	3,853 (61.7)	279 (68.9)	
Overweight	2,330 (33.7)	2,145 (34.4)	185 (27.8)		2,262 (34.0)	2,145 (34.4)	117 (28.9)	
Obesity	257 (3.7)	243 (3.9)	14 (2.1)		252 (3.8)	243 (3.9)	9 (2.2)	
**Smoking**				0.402				0.265
Never	3,233 (46.8)	2,916 (46.7)	317 (47.6)		3,121 (47.0)	2,916 (46.7)	205 (50.6)	
Former	1,868 (27.0)	1,702 (27.3)	166 (24.9)		1,800 (27.1)	1,702 (27.3)	98 (24.2)	
Current	1,806 (26.1)	1,623 (26.0)	183 (27.5)		1,725 (26.0)	1,623 (26.0)	102 (25.2)	
**Drinking (days/week)**				<0.05				<0.05
<1	3,700 (53.6)	3,308 (53.0)	392 (58.9)		3,550 (53.4)	3,308 (53.0)	242 (59.8)	
1-3	2,417 (35.0)	2,226 (35.7)	191 (28.7)		2,345 (35.3)	2,226 (35.7)	119 (29.4)	
4-6	529 (7.7)	481 (7.7)	48 (7.2)		506 (7.6)	481 (7.7)	25 (6.2)	
7	261 (3.8)	226 (3.6)	35 (5.3)		245 (3.7)	226 (3.6)	19 (4.7)	
**Tumor Location**				<0.001				<0.001
Fundus	30 (0.4)	264 (4.2)	4 (0.6)		29 (0.4)	26 (0.4)	3 (0.7)	
Body	2,918 (42.2)	2,625 (42.1)	293 (44.0)		2,809 (42.3)	2,625 (42.1)	184 (45.4)	
Antrum	3,290 (47.6)	3,016 (48.3)	274 (41.1)		3,168 (47.7)	3,016 (48.3)	152 (37.5)	
Pylorus	91 (1.3)	76 (1.2)	15 (2.3)		88 (1.3)	76 (1.2)	12 (3.0)	
Others	578 (8.4)	498 (8.0)	80 (12.0)		552 (8.3)	498 (8.0)	54 (13.3)	
**CEA**	25 ± 24.6	24 ± 22.6	33 ± 37.2	<0.001	25 ± 24.3	24 ± 22.6	34 ± 41.4	<0.001
**CA19-9**	88 ± 63.9	87 ± 63.3	95 ± 68.9	<0.05	88 ± 63.9	87 ± 63.3	97 ± 72.0	<0.05
**Tumor Size**	30 ± 24.8	28 ± 21.4	49 ± 41.1	<0.001	30 ± 24.7	28 ± 21.4	61 ± 44.7	<0.001
**Lymph Node Count**				<0.001				<0.001
0	3,698 (53.5)	3,465 (55.5)	233 (35.0)		3,542 (53.3)	3,465 (55.5)	77 (19.0)	
1-6	817 (11.8)	703 (11.3)	114 (17.1)		789 (11.9)	703 (11.3)	86 (21.2)	

7-15	245 (3.5)	177 (2.8)	68 (10.2)		234 (3.5)	177 (2.8)	57 (14.1)	
≥16	2,147 (31.1)	1,896 (30.4)	251 (37.7)		2,081 (31.3)	1,896 (30.4)	185 (45.7)	
**AJCC7 STAGE**				<0.001				<0.001
I	5,543 (80.3)	5,263 (84.3)	280 (42.0)		5,338 (80.3)	5,263 (84.3)	75 (18.5)	
II	682 (9.9)	586 (9.4)	96 (14.4)		652 (9.8)	586 (9.4)	66 (16.3)	
III	363 (5.3)	264 (4.2)	99 (14.9)		352 (5.3)	264 (4.2)	88 (21.7)	
IV	319 (4.6)	128 (2.1)	191 (28.7)		304 (4.6)	128 (2.1)	176 (43.5)	
**GRADE**				<0.001				<0.001
1	1,819 (26.3)	1,720 (27.6)	99 (14.9)		1,749 (26.3)	1,720 (27.6)	29 (7.2)	
2	2,599 (37.6)	2,369 (38.0)	230 (34.5)		2,490 (37.5)	2,369 (38.0)	121 (29.9)	
3	2,489 (36.0)	2,152 (34.5)	337 (50.6)		2,407 (36.2)	2,152 (34.5)	255 (63.0)	
**Physical activity (days/week)**				0.485				0.88
<1	2,581 (37.4)	2,327 (37.3)	254 (38.1)		2,481 (37.3)	2,327 (37.3)	154 (38.0)	
1-3	3,543 (51.3)	3,197 (51.2)	346 (52.0)		3,406 (51.2)	3,197 (51.2)	209 (51.6)	
4-5	598 (8.7)	551 (8.8)	47 (7.1)		582 (8.8)	551 (8.8)	31 (7.7)	
≥6	185 (2.7)	166 (2.7)	19 (2.9)		177 (2.7)	166 (2.7)	11 (2.7)	
**Check-up repetition**				<0.05				<0.05
Never	1,423 (20.6)	1,237 (19.8)	186 (27.9)		1,366 (20.6)	1,237 (19.8)	129 (31.9)	
1-2	2,981 (43.2)	2,642 (42.3)	339 (50.9)		2,825 (42.5)	2,642 (42.3)	183 (45.2)	
3-4	1,435 (20.8)	1,326 (21.2)	109 (16.4)		1,397 (21.0)	1,326 (21.2)	71 (17.5)	
5-6	804 (11.6)	777 (12.4)	27 (4.1)		794 (11.9)	777 (12.4)	17 (4.2)	
7-8	168 (2.4)	166 (2.7)	2 (0.3)		168 (2.5)	166 (2.7)	2 (0.5)	
9-10	96 (1.4)	93 (1.5)	3 (0.5)		96 (1.4)	93 (1.5)	3 (0.7)	
**Insurance**				0.22				0.848
Medical aids	131 (1.9)	114 (1.8)	17 (2.6)		123 (1.9)	114 (1.8)	9 (2.2)	
Self-employed insured	2,034 (29.4)	1,852 (29.7)	182 (27.3)		1,972 (29.7)	1,852 (29.7)	120 (29.6)	
Employee insured	4,742 (68.7)	4,275 (68.5)	467 (70.1)		4,551 (68.5)	4,275 (68.5)	276 (68.1)	
**Residence**				<0.05				0.29
Urban	3,183 (46.1)	2,912 (46.7)	271 (40.7)		3,090 (46.5)	2,912 (46.7)	178 (44.0)	
Rural	3,724 (53.9)	3,329 (53.3)	395 (59.3)		3,556 (53.5)	3,329 (53.3)	227 (56.0)	
**Income**				0.695				0.784
Low	1,165 (16.9)	1,048 (16.8)	117 (17.6)		1,122 (16.9)	1,048 (16.8)	74 (18.3)	
Middle low	1,527 (22.1)	1,377 (22.1)	150 (22.5)		1,469 (22.1)	1,377 (22.1)	92 (22.7)	
Middle high	2,120 (30.7)	1,929 (30.9)	191 (28.7)		2,046 (30.8)	1,929 (30.9)	117 (28.9)	

	High	2,095 (30.3)	1,887 (30.2)	208 (31.2)		2,009 (30.2)	1,887 (30.2)	122 (30.1)	
**Disability**					0.083				0.766
	No	6,217 (90.0)	5,633 (90.3)	584 (87.7)		6,003 (90.3)	5,633 (90.3)	370 (91.4)	
	Severe	190 (2.8)	170 (2.7)	20 (3.0)		180 (2.7)	170 (2.7)	10 (2.5)	
	Mild	500 (7.2)	438 (7.0)	62 (9.3)		463 (7.0)	438 (7.0)	25 (6.2)	
**Stroke**					<0.001				0.159
	No	6,744 (97.6)	6,197 (99.3)	637 (95.6)		6,499 (97.8)	6,107 (97.9)	386 (95.3)	
	Yes	163 (2.4)	134 (2.1)	29 (4.4)		303 (4.6)	284 (4.6)	19 (4.7)	
**Heart disease**					<0.05				0.895
	No	6,578 (95.2)	5,957 (95.4)	621 (93.2)		6,343 (95.4)	5,957 (95.4)	386 (95.3)	
	Yes	329 (4.8)	284 (4.6)	45 (6.8)		303 (4.6)	284 (4.6)	19 (4.7)	
**Hypertension**					0.149				0.975
	No	4,581 (66.3)	4,156 (66.6)	425 (63.8)		4,426 (66.6)	4,156 (66.6)	270 (66.7)	
	Yes	2,326 (33.7)	2,085 (33.4)	241 (36.2)		2,220 (33.4)	2,085 (33.4)	135 (33.3)	
**Diabetes**					<0.05				0.825
	No	5,871 (85.0)	5,326 (85.3)	545 (81.8)		5,670 (85.3)	5,326 (85.3)	344 (84.9)	
	Yes	1,036 (15.0)	915 (14.7)	121 (18.2)		976 (14.7)	915 (14.7)	61 (15.1)	
**Tuberculosis**					0.685				0.27
	No	6,812 (98.6)	6,154 (98.6)	658 (98.8)		6,556 (98.6)	6,154 (98.6)	402 (99.3)	
	Yes	95 (1.4)	87 (1.4)	8 (1.2)		90 (1.4)	87 (1.4)	3 (0.7)	
**Dyslipidemia**					<0.001				<0.05
	No	6,418 (92.9)	5,777 (92.6)	641 (96.2)		6,167 (92.8)	5,777 (92.6)	390 (96.3)	
	Yes	489 (7.1)	464 (7.4)	25 (3.8)		479 (7.2)	464 (7.4)	15 (3.7)	

Abbreviation: BMI, Body Mass Index; CEA, Carcinoembryonic Antigen; CA19-9, CA 19-9 Antigen; AJCC7 STAGE, AJCC Cancer Staging (7th edition); GRADE, Tumor Grade.

Supplementary Table S3. Patients characteristics before propensity score matching in biennial for all-cause mortality group.

		2012-2013 (N=1,362)	2014-2015 (N=1,807)	2016-2017 (N=1,786)	2018-2019 (N=1,952)	***P***
**AGE**		61.39±10.27	62.19±10.53	62.61±10.62	63.17±10.66	<0.001
	<50	185 (13.6)	214 (11.8)	202 (11.3)	202 (10.3)	
	50-65	603 (44.3)	780 (43.2)	805 (45.1)	856 (43.9)	0.0874
	≥ 65	574 (42.1)	813 (45.0)	779 (43.6)	894 (45.8)	
**SEX**						0.1275
	Female	364 (26.7)	534 (29.6)	521 (29.2)	595 (30.5)	
	Male	998 (73.3)	1,273 (70.4)	1,265 (70.8)	1,357 (69.5)	
**BMI**						<0.001
	Normal	917 (67.3)	1,149 (63.6)	1,101 (61.6)	1,153 (59.1)	
	Overweight	409 (30.0)	596 (33.0)	611 (34.2)	714 (36.6)	
	Obesity	36 (2.6)	62 (3.4)	74 (4.1)	85 (4.4)	
**Smoking**						0.9906
	Never	633 (46.5)	855 (47.3)	832 (46.6)	913 (46.8)	
	Former	375 (27.5)	491 (27.2)	479 (26.8)	523 (26.8)	
	Current	354 (26.0)	461 (25.5)	475 (26.6)	516 (26.4)	
**Drinking (days/week)**						<0.001
	<1	701 (51.5)	942 (52.1)	922 (51.6)	1,135 (58.1)	
	1-3	469 (34.4)	652 (36.1)	665 (37.2)	631 (32.3)	
	4-6	132 (9.7)	140 (7.7)	126 (7.1)	131 (6.7)	
	7	60 (4.4)	73 (4.0)	73 (4.1)	55 (2.8)	
**Tumor Location**						0.0663
	Fundus	7 (0.5)	7 (0.4)	6 (0.3)	10 (0.5)	
	Body	568 (41.7)	728 (40.3)	792 (44.3)	830 (42.5)	
	Antrum	665 (48.8)	916 (50.7)	794 (44.5)	915 (46.9)	
	Pylorus	21 (1.5)	19 (1.1)	24 (1.3)	27 (1.4)	
	Others	101 (7.4)	137 (7.6)	170 (9.5)	170 (8.7)	
**CEA**						
**CA19-9**						
**Tumor Size**		29.62±25.49	29.82±22.85	29.95±25.36	30.51±25.54	0.7418
**Lymph Node Count**						<0.001
	0	834 (61.2)	968 (53.6)	923 (51.7)	973 (49.8)	
	1-6	178 (13.1)	214 (11.8)	204 (11.4)	221 (11.3)	

7-15	57 (4.2)	62 (3.4)	58 (3.2)	68 (3.5)	
≥16	293 (21.5)	563 (31.2)	601 (33.7)	690 (35.3)	
**AJCC7 STAGE**					0.1879
I	1,085 (79.7)	1,465 (81.1)	1,440 (80.6)	1,553 (79.6)	
II	141 (10.4)	163 (9.0)	184 (10.3)	194 (9.9)	
III	83 (6.1)	96 (5.3)	72 (4.0)	112 (5.7)	
IV	53 (3.9)	83 (4.6)	90 (5.0)	93 (4.8)	
**GRADE**					0.4729
1	369 (27.1)	487 (27.0)	456 (25.5)	507 (26.0)	
2	479 (35.2)	683 (37.8)	684 (38.3)	753 (38.6)	
3	514 (37.7)	637 (35.3)	646 (36.2)	692 (35.5)	
**Physical activity (days/week)**					<0.001
<1	505 (37.1)	609 (33.7)	614 (34.4)	853 (43.7)	
1-3	688 (50.5)	960 (53.1)	955 (53.5)	940 (48.2)	
4-5	122 (9.0)	181 (10.0)	165 (9.2)	130 (6.7)	
≥6	47 (3.5)	57 (3.2)	52 (2.9)	29 (1.5)	
**Check-up repetition**					<0.001
Never	165 (12.1)	437 (24.2)	413 (23.1)	408 (20.9)	
1-2	1,197 (87.9)	1,116 (61.8)	503 (28.2)	165 (8.5)	
3-4	0 (0.0)	254 (14.1)	672 (37.6)	509 (26.1)	
5-6	0 (0.0)	0 (0.0)	198 (11.1)	606 (31.0)	
7-8	0 (0.0)	0 (0.0)	0 (0.0)	168 (8.6)	
9-10	0 (0.0)	0 (0.0)	0 (0.0)	96 (4.9)	
**Insurance**					<0.05
National aids	21 (1.5)	25 (1.4)	36 (2.0)	49 (2.5)	
Self-employed insured	437 (32.1)	517 (28.6)	533 (29.8)	547 (28.0)	
Employee insured	904 (66.4)	1,265 (70.0)	1,217 (68.1)	1,356 (69.5)	
**Residence**					0.5238
Urban	629 (46.2)	807 (44.7)	840 (47.0)	907 (46.5)	
Rural	733 (53.8)	1,000 (55.3)	946 (53.0)	1,045 (53.5)	
**Income**					0.3561
Low	215 (15.8)	300 (16.6)	286 (16.0)	364 (18.6)	
Middle low	313 (23.0)	385 (21.3)	414 (23.2)	415 (21.3)	
Middle high	426 (31.3)	565 (31.3)	531 (29.7)	598 (30.6)	
High	408 (30.0)	557 (30.8)	555 (31.1)	575 (29.5)	

**Disability**						0.6118
	No	1,217 (89.4)	1,619 (89.6)	1,621 (90.8)	1,760 (90.2)	
	Severe	38 (2.8)	57 (3.2)	39 (2.2)	56 (2.9)	
	Mild	107 (7.9)	131 (7.2)	126 (7.1)	136 (7.0)	
**Stroke**						0.8711
	No	1,332 (97.8)	1,761 (97.5)	1,742 (97.5)	1,909 (97.8)	
	Yes	30 (2.2)	46 (2.5)	44 (2.5)	43 (2.2)	
**Heart disease**						0.1333
	No	1,313 (96.4)	1,721 (95.2)	1,694 (94.8)	1,850 (94.8)	
	Yes	49 (3.6)	86 (4.8)	92 (5.2)	102 (5.2)	
**Hypertension**						0.5755
	No	882 (64.8)	1,211 (67.0)	1,190 (66.6)	1,298 (66.5)	
	Yes	480 (35.2)	596 (33.0)	596 (33.4)	654 (33.5)	
**Diabetes**						<0.05
	No	1,146 (84.1)	1,576 (87.2)	1,520 (85.1)	1,629 (83.5)	
	Yes	216 (15.9)	231 (12.8)	266 (14.9)	323 (16.5)	
**Tuberculosis**						<0.001
	No	1,328 (97.5)	1,790 (99.1)	1,760 (98.5)	1,934 (99.1)	
	Yes	34 (2.5)	17 (0.9)	26 (1.5)	18 (0.9)	
**Dyslipidemia**						<0.05
	No	1,273 (93.5)	1,689 (93.5)	1,671 (93.6)	1,785 (91.4)	
	Yes	89 (6.5)	118 (6.5)	115 (6.4)	167 (8.6)	

Abbreviation: BMI, Body Mass Index; CEA, Carcinoembryonic Antigen; CA19-9, CA 19-9 Antigen; AJCC7 STAGE, AJCC Cancer Staging (7th edition); GRADE, Tumor Grade.

Supplementary Table S4. Patients characteristics after propensity score matching in biennial for all-cause mortality group.

		2012-2013 (N=684)	2014-2015 (N=903)	2016-2017 (N=670)	2018-2019 (N=407)	*P*
**AGE**		66.39±10.28	67.37±11.43	67.85±10.89	68.12±11.42	<0.05
	<50	51 (7.5)	66 (7.3)	44 (6.6)	30 (7.4)	
	50-65	445 (65.1)	590 (65.3)	438 (65.4)	269 (66.1)	0.9939
	≥ 65	188 (27.5)	247 (27.4)	188 (28.1)	108 (26.5)	
**SEX**						<0.001
	Female	154 (22.5)	248 (27.5)	226 (33.7)	127 (31.2)	
	Male	530 (77.5)	655 (72.5)	444 (66.3)	280 (68.8)	
**BMI**						<0.001
	Normal	504 (73.7)	606 (67.1)	477 (71.2)	247 (60.7)	
	Overweight	169 (24.7)	287 (31.8)	185 (27.6)	152 (37.3)	
	Obesity	11 (1.6)	10 (1.1)	8 (1.2)	8 (2.0)	
**Smoking**						<0.05
	Never	299 (43.7)	429 (47.5)	349 (52.1)	203 (49.9)	
	Former	238 (34.8)	293 (32.4)	172 (25.7)	130 (31.9)	
	Current	147 (21.5)	181 (20.0)	149 (22.2)	74 (18.2)	
**Drinking (days/week)**						<0.001
	<1	375 (54.8)	499 (55.3)	428 (63.9)	262 (64.4)	
	1-3	217 (31.7)	321 (35.5)	191 (28.5)	116 (28.5)	
	4-6	64 (9.4)	55 (6.1)	34 (5.1)	21 (5.2)	
	7	28 (4.1)	28 (3.1)	17 (2.5)	8 (2.0)	
**Tumor Location**						<0.05
	Fundus	2 (0.3)	0 (0.0)	1 (0.1)	2 (0.5)	
	Body	301 (44.0)	378 (41.9)	304 (45.4)	164 (40.3)	
	Antrum	325 (47.5)	454 (50.3)	305 (45.5)	190 (46.7)	
	Pylorus	8 (1.2)	5 (0.6)	11 (1.6)	3 (0.7)	
	Others	48 (7.0)	66 (7.3)	49 (7.3)	48 (11.8)	
**CEA**						
**CA19-9**						
**Tumor Size**		36.26±30.72	38.47±26.68	41.70±30.43	52.40±37.44	<0.001
**Lymph Node Count**						<0.001
	0	351 (51.3)	334 (37.0)	236 (35.2)	95 (23.3)	
	1-6	139 (20.3)	180 (19.9)	130 (19.4)	94 (23.1)	

7-15	92 (13.5)	138 (15.3)	99 (14.8)	61 (15.0)	
≥16	102 (14.9)	251 (27.8)	205 (30.6)	157 (38.6)	
**AJCC7 STAGE**					<0.05
I	407 (59.5)	492 (54.5)	325 (48.5)	137 (33.7)	
II	103 (15.1)	106 (11.7)	103 (15.4)	77 (18.9)	
III	114 (16.7)	185 (20.5)	111 (16.6)	85 (20.9)	
IV	60 (8.8)	120 (13.3)	131 (19.6)	108 (26.5)	
**GRADE**					<0.05
1	106 (15.5)	145 (16.1)	77 (11.5)	51 (12.5)	
2	291 (42.5)	370 (41.0)	272 (40.6)	144 (35.4)	
3	287 (42.0)	388 (43.0)	321 (47.9)	212 (52.1)	
**Physical activity (days/week)**					<0.05
<1	236 (34.5)	344 (38.1)	258 (38.5)	178 (43.7)	
1-3	377 (55.1)	484 (53.6)	365 (54.5)	210 (51.6)	
4-5	59 (8.6)	62 (6.9)	36 (5.4)	15 (3.7)	
≥6	12 (1.8)	13 (1.4)	11 (1.6)	4 (1.0)	
**Check-up repetition**					<0.001
Never	93 (13.6)	220 (24.4)	180 (26.9)	151 (37.1)	
1-2	591 (86.4)	589 (65.2)	224 (33.4)	58 (14.3)	
3-4	0 (0.0)	94 (10.4)	231 (34.5)	98 (24.1)	
5-6	0 (0.0)	0 (0.0)	35 (5.2)	81 (19.9)	
7-8	0 (0.0)	0 (0.0)	0 (0.0)	15 (3.7)	
9-10	0 (0.0)	0 (0.0)	0 (0.0)	4 (1.0)	
**Insurance**					<0.05
National aids	9 (1.3)	3 (0.3)	10 (1.5)	8 (2.0)	
Self-employed insured	175 (25.6)	214 (23.7)	201 (30.0)	108 (26.5)	
Employee insured	500 (73.1)	686 (76.0)	459 (68.5)	291 (71.5)	
**Residence**					0.3069
Urban	260 (38.0)	377 (41.7)	277 (41.3)	176 (43.2)	
Rural	424 (62.0)	526 (58.3)	393 (58.7)	231 (56.8)	
**Income**					<0.05
Low	106 (15.5)	118 (13.1)	91 (13.6)	70 (17.2)	
Middle low	184 (26.9)	186 (20.6)	153 (22.8)	98 (24.1)	
Middle high	186 (27.2)	269 (29.8)	228 (34.0)	114 (28.0)	

	High	208 (30.4)	330 (36.5)	198 (29.6)	125 (30.7)	
**Disability**						0.1642
	No	596 (87.1)	820 (90.8)	605 (90.3)	374 (91.9)	
	Severe	30 (4.4)	24 (2.7)	22 (3.3)	10 (2.5)	
	Mild	58 (8.5)	59 (6.5)	43 (6.4)	23 (5.7)	
**Stroke**						0.1795
	No	665 (97.2)	871 (96.5)	656 (97.9)	400 (98.3)	
	Yes	19 (2.8)	32 (3.5)	14 (2.1)	7 (1.7)	
**Heart disease**						0.4434
	No	662 (96.8)	866 (95.9)	645 (96.3)	386 (94.8)	
	Yes	22 (3.2)	37 (4.1)	25 (3.7)	21 (5.2)	
**Hypertension**						0.9884
	No	443 (64.8)	590 (65.3)	440 (65.7)	266 (65.4)	
	Yes	241 (35.2)	313 (34.7)	230 (34.3)	141 (34.6)	
**Diabetes**						<0.001
	No	586 (85.7)	815 (90.3)	597 (89.1)	327 (80.3)	
	Yes	98 (14.3)	88 (9.7)	73 (10.9)	80 (19.7)	
**Tuberculosis**						<0.001
	No	668 (97.7)	902 (99.9)	668 (99.7)	405 (99.5)	
	Yes	16 (2.3)	1 (0.1)	2 (0.3)	2 (0.5)	
**Dyslipidemia**						<0.05
	No	666 (97.4)	869 (96.2)	650 (97.0)	378 (92.9)	
	Yes	18 (2.6)	34 (3.8)	20 (3.0)	29 (7.1)	

Abbreviation: BMI, Body Mass Index; CEA, Carcinoembryonic Antigen; CA19-9, CA 19-9 Antigen; AJCC7 STAGE, AJCC Cancer Staging (7th edition); GRADE, Tumor Grade.

Supplementary Table S5. Patients characteristics before propensity score matching in biennial for disease specific mortality group.

		2012-2013 (N=1,259)	2014-2015 (N=1,724)	2016-2017 (N=1,733)	2018-2019 (N=1,930)	***P***
**AGE**		60.72±10.13	61.78±10.45	62.38±10.56	63.17±10.66	<0.001
	<50	182 (14.5)	213 (12.4)	200 (11.5)	202 (10.5)	
	50-65	586 (46.5)	763 (44.3)	794 (45.8)	851 (44.1)	<0.05
	≥ 65	491 (39.0)	748 (43.4)	739 (42.6)	877 (45.4)	
**SEX**						0.3285
	Female	348 (27.6)	521 (30.2)	510 (29.4)	589 (30.5)	
	Male	911 (72.4)	1,203 (69.8)	1,223 (70.6)	1,341 (69.5)	
**BMI**						<0.05
	Normal	834 (66.2)	1,096 (63.6)	1,065 (61.5)	1,137 (58.9)	
	Overweight	391 (31.1)	566 (32.8)	597 (34.4)	708 (36.7)	
	Obesity	34 (2.7)	62 (3.6)	71 (4.1)	85 (4.4)	
**Smoking**						0.9612
	Never	586 (46.5)	824 (47.8)	807 (46.6)	904 (46.8)	
	Former	350 (27.8)	466 (27.0)	465 (26.8)	519 (26.9)	
	Current	323 (25.7)	434 (25.2)	461 (26.6)	507 (26.3)	
**Drinking (days/week)**						<0.001
	<1	179 (14.2)	255 (14.8)	264 (15.2)	222 (11.5)	
	1-3	93 (7.4)	213 (12.4)	154 (8.9)	103 (5.3)	
	4-6	34 (2.7)	27 (1.6)	17 (1.0)	15 (0.8)	
	7	10 (0.8)	15 (0.9)	12 (0.7)	7 (0.4)	
**Tumor Location**						0.0537
	Fundus	6 (0.5)	7 (0.4)	6 (0.3)	16 (0.8)	
	Body	527 (41.9)	693 (40.2)	770 (44.4)	10 (0.5)	
	Antrum	615 (48.8)	876 (50.8)	771 (44.5)	819 (42.4)	
	Pylorus	21 (1.7)	19 (1.1)	22 (1.3)	906 (46.9)	
	Others	90 (7.1)	129 (7.5)	164 (9.5)	26 (1.3)	
**CEA**						
**CA19-9**						
**Tumor Size**		29.73 ± 26.07	29.65 ± 22.16	29.95 ± 25.55	30.38 ± 25	0.8214
**Lymph Node Count**						<0.001
	0	763 (60.6)	925 (53.7)	891 (51.4)	963 (49.9)	
	1-6	169 (13.4)	204 (11.8)	199 (11.5)	217 (11.2)	

7-15	51 (4.1)	60 (3.5)	56 (3.2)	67 (3.5)	
≥16	276 (21.9)	535 (31.0)	587 (33.9)	683 (35.4)	
**AJCC7 STAGE**					0.1854
I	1,002 (79.6)	1,398 (81.1)	1,399 (80.7)	1,539 (79.7)	
II	128 (10.2)	155 (9.0)	176 (10.2)	193 (10.0)	
III	80 (6.4)	93 (5.4)	70 (4.0)	109 (5.6)	
IV	49 (3.9)	78 (4.5)	88 (5.1)	89 (4.6)	
**GRADE**					0.4797
1	342 (27.2)	458 (26.6)	446 (25.7)	503 (26.1)	
2	437 (34.7)	651 (37.8)	662 (38.2)	740 (38.3)	
3	480 (38.1)	615 (35.7)	625 (36.1)	687 (35.6)	
**Physical activity (days/week)**					<0.001
<1	467 (37.1)	571 (33.1)	599 (34.6)	844 (43.7)	
1-3	633 (50.3)	923 (53.5)	923 (53.3)	927 (48.0)	
4-5	114 (9.1)	175 (10.2)	163 (9.4)	130 (6.7)	
≥6	45 (3.6)	55 (3.2)	48 (2.8)	29 (1.5)	
**Check-up repetition**					<0.001
Never	152 (12.1)	417 (24.2)	400 (23.1)	397 (20.6)	
1-2	1,107 (87.9)	1,062 (61.6)	492 (28.4)	164 (8.5)	
3-4	0 (0.0)	245 (14.2)	647 (37.3)	505 (26.2)	
5-6	0 (0.0)	0 (0.0)	194 (11.2)	600 (31.1)	
7-8	0 (0.0)	0 (0.0)	0 (0.0)	168 (8.7)	
9-10	0 (0.0)	0 (0.0)	0 (0.0)	96 (5.0)	
**Insurance**					<0.05
National aids	16 (1.3)	23 (1.3)	35 (2.0)	49 (2.5)	
Self-employed insured	407 (32.3)	503 (29.2)	519 (29.9)	543 (28.1)	
Employee insured	836 (66.4)	1,198 (69.5)	1,179 (68.0)	1,338 (69.3)	
**Residence**					0.6516
Urban	595 (47.3)	781 (45.3)	818 (47.2)	896 (46.4)	
Rural	664 (52.7)	943 (54.7)	915 (52.8)	1,034 (53.6)	
**Income**					0.3188
Low	197 (15.6)	286 (16.6)	280 (16.2)	359 (18.6)	
Middle low	289 (23.0)	372 (21.6)	402 (23.2)	406 (21.0)	
Middle high	404 (32.1)	531 (30.8)	515 (29.7)	596 (30.9)	

	High	369 (29.3)	535 (31.0)	536 (30.9)	569 (29.5)	
**Disability**						0.637
	No	1,133 (90.0)	1,550 (89.9)	1,577 (91.0)	1,743 (90.3)	
	Severe	33 (2.6)	55 (3.2)	37 (2.1)	55 (2.8)	
	Mild	93 (7.4)	119 (6.9)	119 (6.9)	132 (6.8)	
**Stroke**						0.9071
	No	1,233 (97.9)	1,686 (97.8)	1,691 (97.6)	1,889 (97.9)	
	Yes	26 (2.1)	38 (2.2)	42 (2.4)	41 (2.1)	
**Heart disease**						0.0731
	No	1,218 (96.7)	1,646 (95.5)	1,649 (95.2)	1,830 (94.8)	
	Yes	41 (3.3)	78 (4.5)	84 (4.8)	100 (5.2)	
**Hypertension**						0.6984
	No	823 (65.4)	1,163 (67.5)	1,154 (66.6)	1,286 (66.6)	
	Yes	436 (34.6)	561 (32.5)	579 (33.4)	644 (33.4)	
**Diabetes**						<0.05
	No	1,071 (85.1)	1,509 (87.5)	1,475 (85.1)	1,615 (83.7)	
	Yes	188 (14.9)	215 (12.5)	258 (14.9)	315 (16.3)	
**Tuberculosis**						<0.001
	No	1,228 (97.5)	1,707 (99.0)	1,707 (98.5)	1,914 (99.2)	
	Yes	31 (2.5)	17 (1.0)	26 (1.5)	16 (0.8)	
**Dyslipidemia**						0.0549
	No	1,171 (93.0)	1,611 (93.4)	1,620 (93.5)	1,765 (91.5)	
	Yes	88 (7.0)	113 (6.6)	113 (6.5)	165 (8.5)	

Abbreviation: BMI, Body Mass Index; CEA, Carcinoembryonic Antigen; CA19-9, CA 19-9 Antigen; AJCC7 STAGE, AJCC Cancer Staging (7th edition); GRADE, Tumor Grade.

Supplementary Table S6. Patients characteristics after propensity score matching in biennial for disease specific mortality group.

		2012-2013 (N=316)	2014-2015 (N=510)	2016-2017 (N=510)	2018-2019 (N=447)	*P*
**AGE**		63.79±11.52	64.82±12.54	65.54±11.93	67.98±11.51	<0.001
	<50	37 (11.7)	56 (11.0)	50 (11.2)	25 (7.2)	
	50-65	112 (35.4)	185 (36.3)	139 (31.1)	94 (27.1)	<0.05
	≥ 65	167 (52.8)	269 (52.7)	258 (57.7)	228 (65.7)	
**SEX**						0.3461
	Female	87 (27.5)	157 (30.8)	141 (31.5)	118 (34.0)	
	Male	229 (72.5)	353 (69.2)	306 (68.5)	229 (66.0)	
**BMI**						<0.05
	Normal	234 (74.1)	334 (65.5)	326 (72.9)	213 (61.4)	
	Overweight	76 (24.1)	169 (33.1)	114 (25.5)	124 (35.7)	
	Obesity	6 (1.9)	7 (1.4)	7 (1.6)	10 (2.9)	
**Smoking**						0.2035
	Never	149 (47.2)	246 (48.2)	208 (46.5)	183 (52.7)	
	Former	105 (33.2)	170 (33.3)	136 (30.4)	110 (31.7)	
	Current	62 (19.6)	94 (18.4)	103 (23.0)	54 (15.6)	
**Drinking (days/week)**						<0.001
	<1	179 (56.6)	255 (50.0)	264 (59.1)	222 (64.0)	
	1-3	93 (29.4)	213 (41.8)	154 (34.5)	103 (29.7)	
	4-6	34 (10.8)	27 (5.3)	17 (3.8)	15 (4.3)	
	7	10 (3.2)	15 (2.9)	12 (2.7)	7 (2.0)	
**Tumor Location**						<0.001
	Fundus	4 (1.3)	0 (0.0)	1 (0.2)	3 (0.9)	
	Body	155 (49.1)	231 (45.3)	222 (49.7)	155 (44.7)	
	Antrum	123 (38.9)	237 (46.5)	177 (39.6)	138 (39.8)	
	Pylorus	3 (0.9)	4 (0.8)	12 (2.7)	2 (0.6)	
	Others	31 (9.8)	38 (7.5)	35 (7.8)	49 (14.1)	
**CEA**						
**CA19-9**						
**Tumor Size**		45.72 ± 38.59	45.11 ± 26.10	50.11 ± 33.85	57 (16.4)	
**Lymph Node Count**						
	0	95 (30.1)	119 (23.3)	98 (21.9)	62 (17.9)	
	1-6	97 (30.7)	137 (26.9)	103 (23.0)	77 (22.2)	

7-15	72 (22.8)	131 (25.7)	101 (22.6)	78 (22.5)	
≥16	52 (16.5)	123 (24.1)	145 (32.4)	130 (37.5)	
**AJCC7 STAGE**					<0.001
I	103 (32.6)	162 (31.8)	128 (28.6)	82 (23.6)	
II	56 (17.7)	70 (13.7)	86 (19.2)	55 (15.9)	
III	101 (32.0)	171 (33.5)	95 (21.3)	97 (28.0)	
IV	56 (17.7)	107 (21.0)	138 (30.9)	113 (32.6)	
**GRADE**					0.3946
1	33 (10.4)	42 (8.2)	34 (7.6)	19 (5.5)	
2	106 (33.5)	186 (36.5)	165 (36.9)	130 (37.5)	
3	177 (56.0)	282 (55.3)	248 (55.5)	198 (57.1)	
**Physical activity (days/week)**					0.0929
<1	100 (31.6)	179 (35.1)	156 (34.9)	152 (43.8)	
1-3	188 (59.5)	294 (57.6)	259 (57.9)	178 (51.3)	
4-5	22 (7.0)	30 (5.9)	28 (6.3)	14 (4.0)	
≥6	6 (1.9)	7 (1.4)	4 (0.9)	3 (0.9)	
**Check-up repetition**					<0.001
Never	59 (18.7)	137 (26.9)	129 (28.9)	110 (31.7)	
1-2	257 (81.3)	290 (56.9)	133 (29.8)	38 (11.0)	
3-4	0 (0.0)	83 (16.3)	155 (34.7)	86 (24.8)	
5-6	0 (0.0)	0 (0.0)	30 (6.7)	93 (26.8)	
7-8	0 (0.0)	0 (0.0)	0 (0.0)	12 (3.5)	
9-10	0 (0.0)	0 (0.0)	0 (0.0)	8 (2.3)	
**Insurance**					<0.001
National aids	1 (0.3)	1 (0.2)	6 (1.3)	7 (2.0)	
Self-employed insured	76 (24.1)	119 (23.3)	154 (34.5)	96 (27.7)	
Employee insured	239 (75.6)	390 (76.5)	287 (64.2)	244 (70.3)	
**Residence**					0.4538
Urban	135 (42.7)	211 (41.4)	185 (41.4)	161 (46.4)	
Rural	181 (57.3)	299 (58.6)	262 (58.6)	186 (53.6)	
**Income**					0.322
Low	50 (15.8)	70 (13.7)	72 (16.1)	54 (15.6)	
Middle low	83 (26.3)	115 (22.5)	98 (21.9)	74 (21.3)	
Middle high	85 (26.9)	157 (30.8)	157 (35.1)	111 (32.0)	

	High	98 (31.0)	168 (32.9)	120 (26.8)	108 (31.1)	
**Disability**						0.9277
	No	292 (92.4)	475 (93.1)	407 (91.1)	319 (91.9)	
	Severe	9 (2.8)	14 (2.7)	18 (4.0)	12 (3.5)	
	Mild	15 (4.7)	21 (4.1)	22 (4.9)	16 (4.6)	
**Stroke**						0.5412
	No	308 (97.5)	498 (97.6)	436 (97.5)	343 (98.8)	
	Yes	8 (2.5)	12 (2.4)	11 (2.5)	4 (1.2)	
**Heart disease**						0.4225
	No	310 (98.1)	492 (96.5)	429 (96.0)	336 (96.8)	
	Yes	6 (1.9)	18 (3.5)	18 (4.0)	11 (3.2)	
**Hypertension**						0.9845
	No	220 (69.6)	353 (69.2)	306 (68.5)	238 (68.6)	
	Yes	96 (30.4)	157 (30.8)	141 (31.5)	109 (31.4)	
**Diabetes**						<0.05
	No	279 (88.3)	457 (89.6)	404 (90.4)	287 (82.7)	
	Yes	37 (11.7)	53 (10.4)	43 (9.6)	60 (17.3)	
**Tuberculosis**						0.0637
	No	312 (98.7)	509 (99.8)	445 (99.6)	347 (100.0)	
	Yes	4 (1.3)	1 (0.2)	2 (0.4)	0 (0.0)	
**Dyslipidemia**						0.3349
	No	305 (96.5)	497 (97.5)	430 (96.2)	330 (95.1)	
	Yes	11 (3.5)	13 (2.5)	17 (3.8)	17 (4.9)	

Abbreviation: BMI, Body Mass Index; CEA, Carcinoembryonic Antigen; CA19-9, CA 19-9 Antigen; AJCC7 STAGE, AJCC Cancer Staging (7th edition); GRADE, Tumor Grade.
