# Supplementary material for: Predictive Mortality and Gastric Cancer Risk Using Clinical and Socio-Economic Data: A Nationwide Multicenter Cohort Study
Source: Cancers (Basel). 2024 Dec 25;17(1):30. doi: 10.3390/cancers17010030 (PMC11718814; doi:10.3390/cancers17010030)
Supplement: Supplementary file 1 [file cancers-17-00030-s001.zip › Supplementary_figures.pdf]

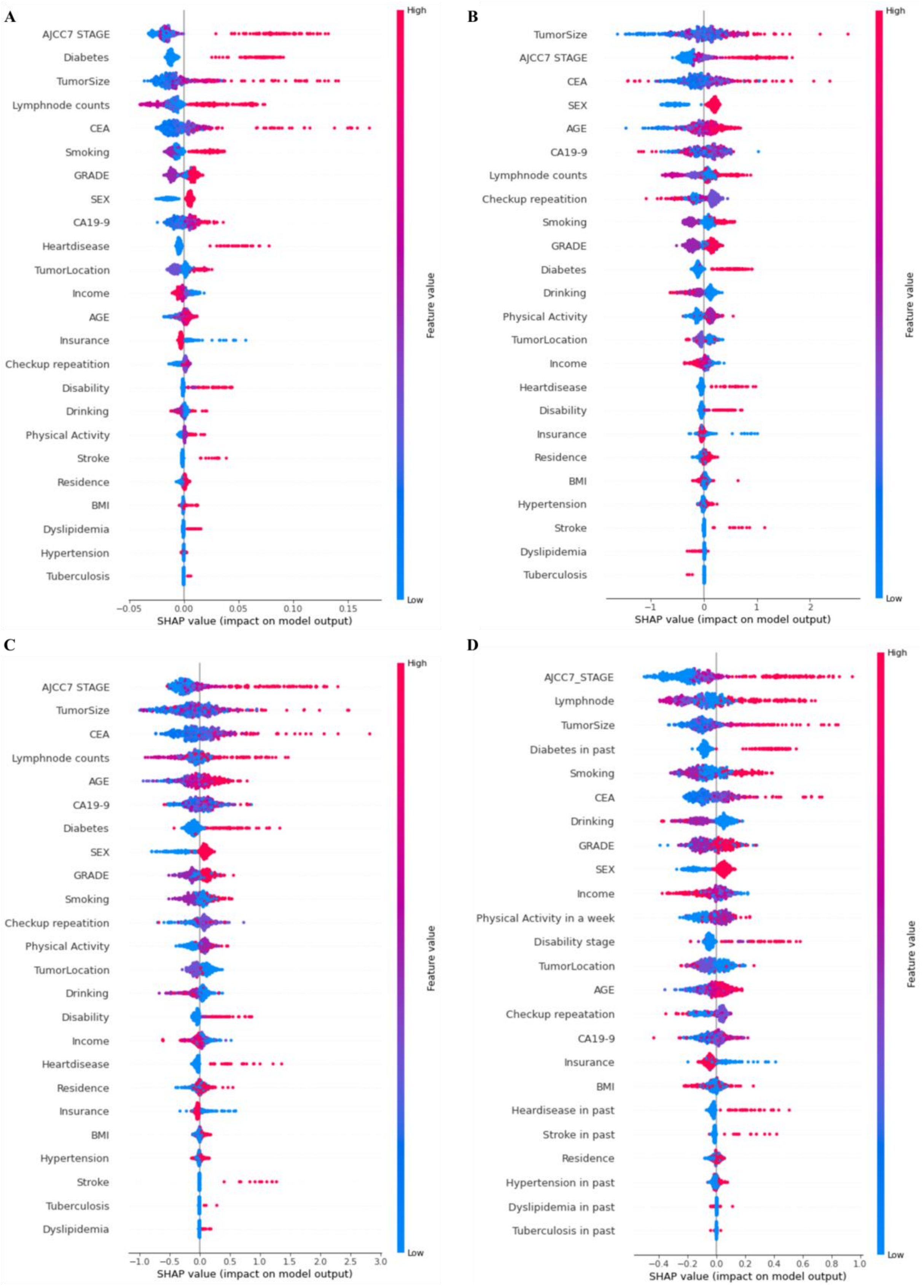


**Supplementary Figure S1. Variables interpretation by SHAP for all-cause mortality. (A)** Random Forest **(B)** Light Gradient Boosting Machine **(C)** Extreme Gradient Boosting **(D)** Categorical Boosting.

The common factors by SHAP interpretation revealed that the AJCC 7th stage, lymph node counts, tumor size, CEA levels, and CA19-9 levels had the most significant impact in relation to mortality risk. Abbreviation: BMI, Body Mass Index; CEA, Carcinoembryonic Antigen; CA19-9, CA 19-9 Antigen; AJCC7 STAGE, AJCC Cancer Staging (7th edition); GRADE, Tumor Grade;

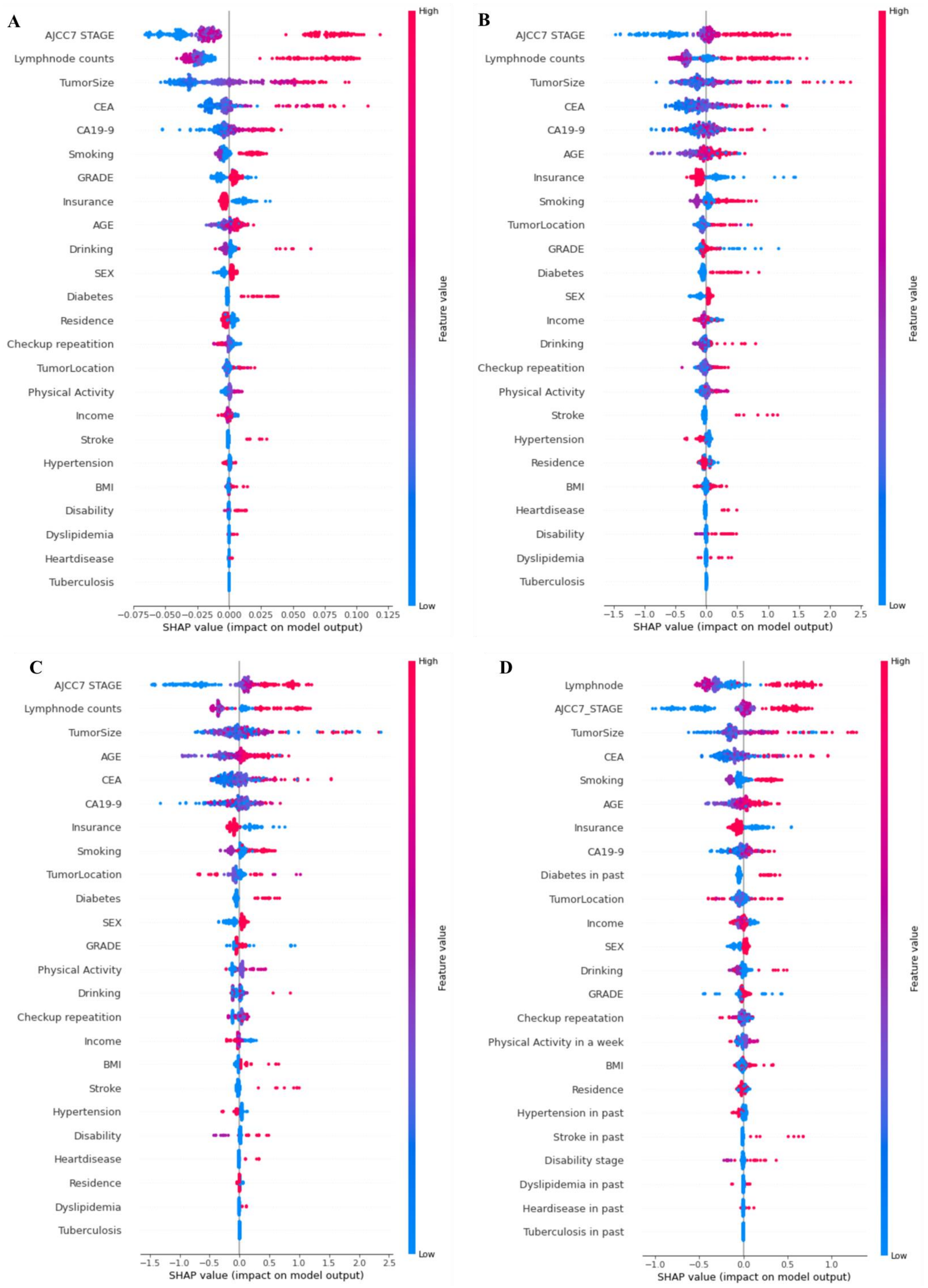


**Supplementary Figure S2. Variables interpretation by SHAP for disease-specific mortality. (A)** Random Forest **(B)** Gradient Boosting Machine **(C)** Extreme Gradient Boosting **(D)** Categorical Boosting.

The common factors by SHAP interpretation revealed that AJCC 7th stage, diabetes, tumor size, lymph node counts, and CEA levels had the most significant impact in relation to mortality risk. Abbreviation: BMI, Body Mass Index; CEA, Carcinoembryonic Antigen; CA19-9, CA 19-9 Antigen; AJCC7 STAGE, AJCC Cancer Staging (7th edition); GRADE, Tumor Grade;
